# Extensive remodeling of DC function by rapid maturation-induced transcriptional silencing

**DOI:** 10.1093/nar/gku674

**Published:** 2014-08-07

**Authors:** Queralt Seguín-Estévez, Isabelle Dunand-Sauthier, Sylvain Lemeille, Christian Iseli, Mark Ibberson, Vassilios Ioannidis, Christoph D. Schmid, Philippe Rousseau, Emmanuèle Barras, Antoine Geinoz, Ioannis Xenarios, Hans Acha-Orbea, Walter Reith

**Affiliations:** 1 Department of Pathology and Immunology, University of Geneva Medical School, CH-1211 Geneva, Switzerland; 2 Vital-IT, Swiss Institute of Bioinformatics, CH-1015 Lausanne, Switzerland; 3 Department of Medical Parasitology and Infection Biology, Swiss Tropical and Public Health Institute, CH-4051 Basel, Switzerland; 4 University of Basel, CH-4051 Basel, Switzerland; 5 Department of Biochemistry, Faculty of Biology and Medicine, University of Lausanne, CH-1066 Epalinges, Switzerland

## Abstract

The activation, or maturation, of dendritic cells (DCs) is crucial for the initiation of adaptive T-cell mediated immune responses. Research on the molecular mechanisms implicated in DC maturation has focused primarily on inducible gene-expression events promoting the acquisition of new functions, such as cytokine production and enhanced T-cell-stimulatory capacity. In contrast, mechanisms that modulate DC function by inducing widespread gene-silencing remain poorly understood. Yet the termination of key functions is known to be critical for the function of activated DCs. Genome-wide analysis of activation-induced histone deacetylation, combined with genome-wide quantification of activation-induced silencing of nascent transcription, led us to identify a novel inducible transcriptional-repression pathway that makes major contributions to the DC-maturation process. This silencing response is a rapid primary event distinct from repression mechanisms known to operate at later stages of DC maturation. The repressed genes function in pivotal processes—including antigen-presentation, extracellular signal detection, intracellular signal transduction and lipid-mediator biosynthesis—underscoring the central contribution of the silencing mechanism to rapid reshaping of DC function. Interestingly, promoters of the repressed genes exhibit a surprisingly high frequency of PU.1-occupied sites, suggesting a novel role for this lineage-specific transcription factor in marking genes poised for inducible repression.

## INTRODUCTION

Dendritic cells (DCs) are professional antigen (Ag) presenting cells playing central roles in the initiation, regulation and implementation of Ag-specific immune responses (1,2). They serve as interfaces between innate and adaptive immunity by promoting the development of appropriate T cell responses in response to signals associated with infection, including pathogen associated molecular patterns (PAMPs), endogenous danger signals (DAMPs) and inflammatory signals. Exposure of DCs to these stimuli triggers a maturation process involving multiple morphological, phenotypic and functional changes (1,3). Major changes include increased Major Histocompatibility Complex class II (MHCII) expression, increased Ag-presentation, enhanced costimulatory-molecule expression, potentiated T-cell-stimulatory capacity, the production of pro-inflammatory and/or anti-inflammatory mediators, and altered migratory properties (1,3).

DCs detect PAMPs and DAMPs via pattern recognition receptors (PRRs), including Toll-like receptors (TLRs), Nod-like receptors (NLRs), C-type lectin receptors (CLRs) and Rig-I-like receptors (RLRs) (4,5). PRR-engagement activates well-defined signaling cascades that induce complex transcriptional responses adapted to the stimuli that elicited them (6–9). Current knowledge on regulatory networks governing transcriptional responses in DCs is largely derived from genome-wide transcriptomic, epigenetic and transcription factor (TF) binding studies performed with mouse bone marrow derived DCs (BM-DCs), and primary cells or cell lines belonging to the monocyte-macrophage lineage (6–9). These studies focused primarily on transcriptional activation. Current models propose that PRR-induced signaling converges on signal-activated TFs, sometimes referred to as class I TFs, including members of the NF-kB, IRF and AP1 families (10,11). Class I TFs induce primary-response genes in a rapid (1–2 h) and protein-synthesis independent manner (10,11). Primary-response genes include genes encoding TFs (class II TFs) governing subsequent waves of transcriptional responses (10–13). Class II TFs induce secondary responses and/or modulate primary responses. Class I and II TFs collaborate with cell-type-specific TFs (class III TFs), certain of which—such as PU-1, RUNX1 or C/EBPβ—are believed to function as ‘pioneer’ factors that establish an accessible chromatin environment at regulatory elements of inducible genes (8,10,11,14–16).

Compared to current knowledge on TF networks governing transcriptional activation in DCs, little is known about mechanisms controlling maturation-induced transcriptional silencing. Certain class II TFs—such as ATF3, BCL6 and PRDM1—function as repressors that dampen or terminate transcriptional responses in activated DCs (17–19). However, virtually nothing is known about primary-response mechanisms mediating silencing during early stages of DC maturation. Yet large-scale expression-profiling studies have indicated that numerous genes are down-regulated rapidly in response to maturation stimuli (7,8). Mechanisms underpinning this large-scale gene-silencing have not been investigated. One reason for this is that our general comprehension of mechanisms regulating inducible gene-silencing is remarkably poor relative to those governing transcriptional activation. Another reason is that mRNA-stability issues confound the temporal analysis of transcriptional silencing by mRNA-expression profiling approaches.

Silencing of specific genes is known to be critical for the function of mature DCs. Notable examples are provided by MHCII-mediated Ag-presentation: silencing of the ubiquitin-ligase MARCH1 stabilizes cell-surface MHCII expression (20) whereas silencing of the MHCII-transactivator CIITA aborts *de novo* MHCII synthesis (21). These processes promote cell-surface retention of peptide-MHCII complexes presenting Ags captured prior to DC-activation. To study maturation-induced repression in DCs, we first focused on the *CIITA* gene. *CIITA* silencing was found to be a highly sensitive primary response triggered in human and mouse DCs by diverse maturation signals, and involves rapid histone-deacetylation over a large chromatin domain. Genes subjected to the same silencing mechanism were next identified by combining genome-wide analyses of histone-deacetylation with a global quantification of nascent transcripts. This identified numerous genes undergoing chromatin-deacetylation and transcriptional-arrest within 1 h. Promoters of these genes are strongly enriched in PU.1-binding sites, suggesting a new role for this TF in marking genes poised for repression. The repressed genes are implicated in key functions—including Ag capture and presentation, extracellular-signal detection, signal transduction and lipid-mediator synthesis—underscoring the pivotal contribution of primary silencing to DC maturation.

## MATERIALS AND METHODS

### Cells

Mo-DCs were generated as described (21). Their maturation was induced with lipopolysaccharide (LPS) (Alexis, 25 ng/ml unless indicated otherwise), polyI:C (Amersham Biosciences, 0.05 mg/ml), peptidoglycan (PGN, Sigma, 10 μg/ml), Pam3CysSerLys4 (PAM3CSK4, InvivoGen, 500 ng/ml), TNFα (InvivoGen, 100 ng/ml) or flagellin (InvivoGen, 200 ng/ml). DC^2114^ cells were activated with CpG as described (22,23). Cells were treated with 165 nM Trichostatin A (TSA, Sigma-Aldrich) or 0.4 mM Cycloheximide (CHX, Sigma-Aldrich). SP600125, SB202190, Lactacystine and U0126 were from Calbiochem.

### qRT-PCR

Total and nascent RNA extractions, and cDNA synthesis, were done as described (24,25). Quantification was done using the iCycler iQ Real-Time polymerase chain reaction (PCR) Detection System (Biorad) and a Sybr-Green-based kit for quantitative PCR (iQ Supermix Biorad). Results were normalized using 18S rRNA. Primer sequences are available upon request.

### Western blotting

Protein extracts were fractionated by Sodium dodecylsulphate-polyacrylamide gel electrophoresis and western blotting was performed using the following antibodies: MARCH1 (Abcam), CLEC10A (Abnova), SOCS5 (GeneTex), CLEC4A (GeneTex) and tubulin (SIGMA-ALDRICH).

### Chromatin immunoprecipitation (ChIP)

ChIP experiments were performed as described (24) using antibodies against H4Ac (Upstate Biotechnology/Millipore), H3Ac (Upstate Biotechnology/Millipore), H3K4trim (Abcam), RNA pol II (Abcam) and PU-1 (Santa Cruz). Results were quantified by real-time PCR using the iCycler iQ Real-Time PCR Detection System (Biorad) and a Sybr-Green-based kit for quantitative PCR (iQ Supermix Biorad). Primer sequences are available upon request.

### ChIP-chip experiments

Three biological replicates of H4Ac-ChIP samples were prepared from immature Mo-DCs and Mo-DCs stimulated for 1 h with LPS, and verified by quantitative PCR to assess LPS-induced H4-deacetylation at the *CIITA* locus. DNA was purified, amplified by LPMCR as described (24) and sent to Roche-NimbleGen for probe preparation and hybridization to HG18 arrays carrying promoter regions (∼−3.5 to +0.75 kb relative to the TSS) of all human genes, or to a custom array of our own design (24). The latter carries unique sequences from the entire extended human MHC locus (7.7 Mb on chromosome 6, genomic coordinates 26.1 to 33.8 Mb in hg17) and selected control regions (total 0.9 Mb). Genomic loci are covered at high density with overlapping Tm-matched oligonucleotides (∼50 bp long) spaced such that their 5′ ends are ∼10 bp apart. Data derived from the promoter arrays was analyzed for peaks using NimbleScan software.

### Nascent RNA extraction

Nascent RNA was isolated essentially as described (21,25). 10–20 × 10^6^ Mo-DCs were washed thrice with phosphate bufferedsaline and resuspended in 10% glycerol, 0.3 M sucrose, 60 mM KCl, 15 mM NaCl, 15 mM HEPES (pH 7.9), 0.5 mM Ethylenediaminetetraacetic acid (EDTA), 0.15 mM Spermine, 0.5 mM Spermidine, 0.5 mM PMSF, 1 mM Dithiothreitol (DTT). Cells were lysed for 10 min on ice after adding one volume of the same solution containing 0.8% NP40. Nuclei were pelleted through a 1 ml cushion of the same solution containing 0.9 M sucrose, resuspended in 75 mM NaCl, 20 mM Tris-HCL (pH 7.9), 0.5 mM EDTA 0.125 mM PMSF 0.85 mM DTT, 50% glycerol and lysed for 10 min on ice after adding 8 volumes of 0.3 M NaCl, 20 mM HEPES (pH 7.6), 0.2 mM EDTA, 7.5 mM MgCl_2_, 1 M Urea, 1 mM DTT, 1% NP-40. Chromatin was pelleted by centrifugation and resuspended in 500 μl of 50 mM NaAc (pH5), 50 mM NaCl, 0.5% sodium dodecyl sulphate (SDS). Nascent RNA was purified by three extractions with hot phenol (saturated with 50 mM NaAc pH5, 50mM NaCl) and precipitated with 0.15 M NaCl and ethanol. RNA was resuspended in 50 μl 10 mM Tris-HCL (pH 7.6) and treated with DNaseI for 15 min at 37°C in 10 mM MgCl_2_, 1 mM DTT. DNase activity was stopped by adding 50 mM EDTA, 1.5 M NaAc, 1% SDS and RNA was purified by phenol-chloroform extraction and ethanol precipitation. Nascent RNA samples were depleted of ribosomal RNA (rRNA) using the Ribominus kit (Invitrogen). Nascent RNA samples corresponding to three independent experiments were subjected to quality controls by qRT-PCR. DNA contamination was excluded by omitting reverse transcriptase. Maturation was assessed by measuring IL12B, IL1B and TNF mRNAs induction. Enrichment of primary transcripts was verified using primers specific for selected unspliced precursor RNAs. rRNA depletion (typically >95%) was verified using primers for 18 and 28S RNAs.

### RNA sequencing

cDNA libraries were generated from three independent nascent RNA or mRNA samples using Illumina specifications. Illumina technology was used to generate paired-end reads (80 bp) for nascent RNA or single-end reads (50 bp) for mRNA. Data analysis consisted of a filtering step to eliminate reads consisting mono- and di-nucleotide repeats followed by the identification of exact unique matches using fetchGWI (26). Megablast was used to recover unmatched reads (http://www.ncbi.nlm.nih.gov/blast/html/megablast.html). Paired information was used to generate alignments of each read on the genomic region where hits were found by previous steps using SIBsim4 http://sibsim4.sourceforge.net/. Alignments were analyzed using Tromer (27).

### ChIP sequencing

10 ng of immunoprecipitated-DNA or chromatin-input-DNA were used to prepare sequencing libraries using Illumina specifications. 50-nucleotide single-end reads were generated using Illumina technology. Reads were aligned to the reference human genome GRCh37.71 using Bowtie version 0.12.7 (http://bowtie-bio.sourceforge.net/index.shtml). Only uniquely mapped reads were considered for further analysis. Peaks were called using MACS software version 2.0.10.20130520 (http://liulab.dfci.harvard.edu/MACS/). Input-DNA was used as background control. Peaks were considered significant if their *P*-values were <10^−^^5^ and if present (overlapping by >1 nt) in two biological repeats.

### TFBS enrichment

Pscan was used to identify potential TF-binding-sites (TFBSs) (JASPAR database) that are overrepresented between nucleotides −450 and +50 relative to the TSS (http://159.149.160.51/pscan/). TFBSs with *P*-values <10^−^^3^ were considered to be significantly overrepresented.

### Gene ontology

Gene ontology analyses were performed at http://david.abcc.ncifcrf.gov/ and http://cbl-gorilla.cs.technion.ac.il/.

## RESULTS

### Silencing of *CIITA*

**Figure 1.**
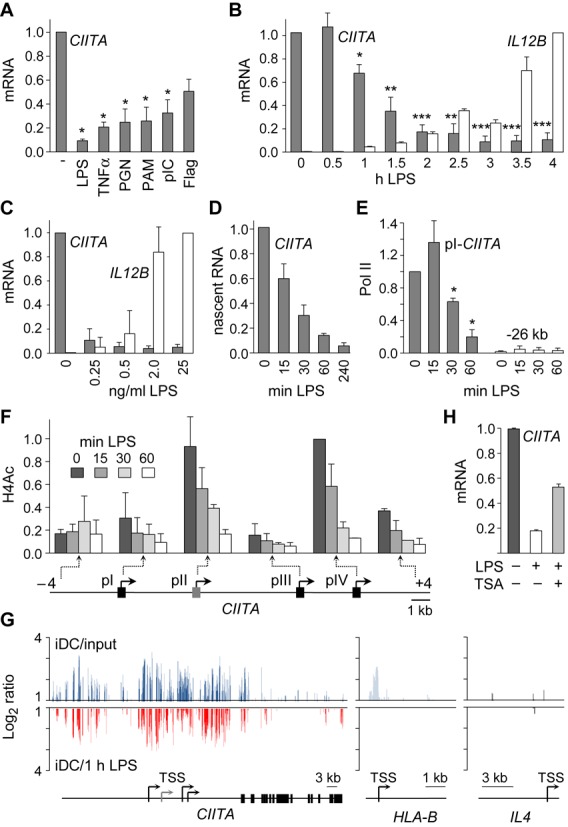
Transcriptional silencing of *CIITA* during DC maturation. (**A**) CIITA mRNA was quantified in Mo-DCs exposed for 24 h to LPS, TNFα, PGN, PAM, pIC or Flagellin. Results are represented relative to unstimulated DCs. Statistical significance was derived from three experiments: *, *P* < 0.05 (**B**) CIITA and IL12B mRNAs were quantified in Mo-DC treated with LPS for the indicated times. Results are represented relative to unstimulated DCs. Statistical significance was derived from three experiments: *, *P* < 0.05, **, *P* < 0.01, ***, *P* < 0.001. (**C**) CIITA and IL12B mRNAs were quantified in Mo-DCs treated for 6 h with the indicated LPS concentrations. Results are expressed relative to unstimulated DCs. Results are derived from two experiments. (**D**) Nascent CIITA transcripts were quantified in Mo-DCs exposed to LPS for the indicated times. Results are expressed relative to unstimulated DCs. Results are derived from two experiments. The data is representative of four experiments. (**E**) Binding of Pol-II to *CIITA* promoter I and a 26 kb upstream region (background control) was assessed by qChIP in Mo-DCs exposed to LPS for the indicated times. Results are expressed relative to immature DCs at *CIITA* promoter I. Statistical significance was derived from three experiments: *, *P* < 0.05. (**F**) H4Ac was measured in Mo-DCs activated with LPS for the indicated times at the indicated positions of *CIITA*. Results are expressed relative to H4Ac at promoter IV in immature DCs. Results are derived from two experiments. The data is representative of four experiments. (**G**) H4Ac-profiling at the *CIITA*, *HLA-B* and *IL4* genes was performed by ChIP-chip. H4Ac in untreated Mo-DCs (blue) was determined as the signal ratio between immature DCs (iDC) and input DNA. H4-deacetylation (red) was determined as the signal ratio between iDCs and DCs exposed to LPS for 1 h. Ratios are represented on a log_2_ scale. Maps of the genes are shown below: the scale in kb and TSSs are indicated. (**H**) CIITA mRNA was quantified in Mo-DCs treated with LPS for 4 h in the absence or presence of TSA. Results are expressed relative to immature DCs. Results are derived from two experiments. The data is representative of four experiments. All measurements were performed in triplicate for each experiment.

CIITA mRNA abundance is decreased during DC maturation (19,21,28). To clarify the mechanism involved, *CIITA*-silencing was investigated in human monocyte-derived DCs (Mo-DCs). *CIITA*-silencing was induced by diverse stimuli—including LPS, tumor necrosis factor (TNFα), peptidoglycan (PGN), Pam3CysSerLys4 (PAM), polyinosinic-polycytidylic acid (pIC) and flagellin (Flag)—indicating that it is a general feature of Mo-DC maturation (Figure 1A). Time-course experiments demonstrated that LPS-induced down-regulation of CIITA mRNA was detectable by 1 h and reached baseline levels by 2 h (Figure 1B). This decrease preceded the induction of IL12B (Figure 1B) and IL6 (data not shown) mRNAs. LPS-concentrations as low as 0.25 ng/ml were sufficient to trigger *CIITA*-silencing, whereas higher concentrations were required for optimal induction of IL12B (Figure 1C) and IL6 (data not shown) mRNAs. Quantification of chromatin-bound nascent transcripts demonstrated that LPS-induced down-regulation of CIITA mRNA resulted from an arrest in transcription that was evident by 15 min and almost complete after 1 h (Figure 1D). Quantitative chromatin-immunoprecipitation (qChIP) experiments revealed a rapid LPS-induced disengagement of RNA-polymerase-II (pol-II) at the DC-specific promoter (pI) of *CIITA* (Figure 1E).

The contribution of epigenetic mechanisms to *CIITA-*silencing was assessed by qChIP experiments using antibodies against histone modifications characteristic of active or repressed chromatin. Active-chromatin marks examined were histones H3 and H4 acetylation (H3Ac, H4Ac), and H3-lysine-4 trimethylation (H3K4trim). Repressed-chromatin marks examined were H3-lysine-9 dimethylation and H3-lysine-27 trimethylation. Deposition of heterochromatin protein (HP1) was also examined. These chromatin features were assessed in LPS-treated Mo-DCs at strategic positions in the regulatory region of *CIITA*. Strong reductions in H4Ac were observed at all four *CIITA* promoters (pI–pIV) and at a position situated 4 kb downstream of pIV (Figure 1F). H4-deacetylation was evident after 15 min (Figure 1F). A similar, albeit less marked, reduction in H3Ac was observed (Supplementary Figure S1A). H3K4trim was also decreased (Supplementary Figure S1A), but this was a secondary event occurring at later time points (24 h). No significant changes in repressive chromatin marks or HP1 deposition were observed during the same time frame (data not shown). H4-deacetylation was thus the most characteristic change associated temporally with *CIITA*-silencing.

ChIP-on-microarray (ChIP-chip) experiments were performed to analyze H4-deacetylation at the *CIITA* locus in greater detail. H4Ac-ChIP samples prepared from immature Mo-DCs and 1 h-LPS-treated Mo-DCs were used to probe a custom-made high-density array carrying the entire *CIITA* locus and control genes (24). Input DNA was used as control. The immature-DC/input-DNA signal ratio was used to assess the initial pattern of H4Ac in immature Mo-DCs. H4-deacetylation was assessed by determining the immature-DC/LPS-treated-DC signal ratio. 1 h of LPS treatment induced removal of the H4Ac mark over a large 40–50 kb region spanning the entire regulatory region of *CIITA* (Figure 1G). Deacetylation was not observed at control genes, such as *HLA-B*, which is not silenced, or the non-expressed *IL4* gene (Figure 1G).

Functional relevance of histone deacetylation for *CIITA* silencing was investigated by using the histone-deacetylase (HDAC) inhibitor Trichostatin A (TSA). TSA impaired LPS-induced CIITA mRNA down-regulation, suggesting that *CIITA* silencing requires histone deacetylation (Figure 1H).

**Figure 2.**
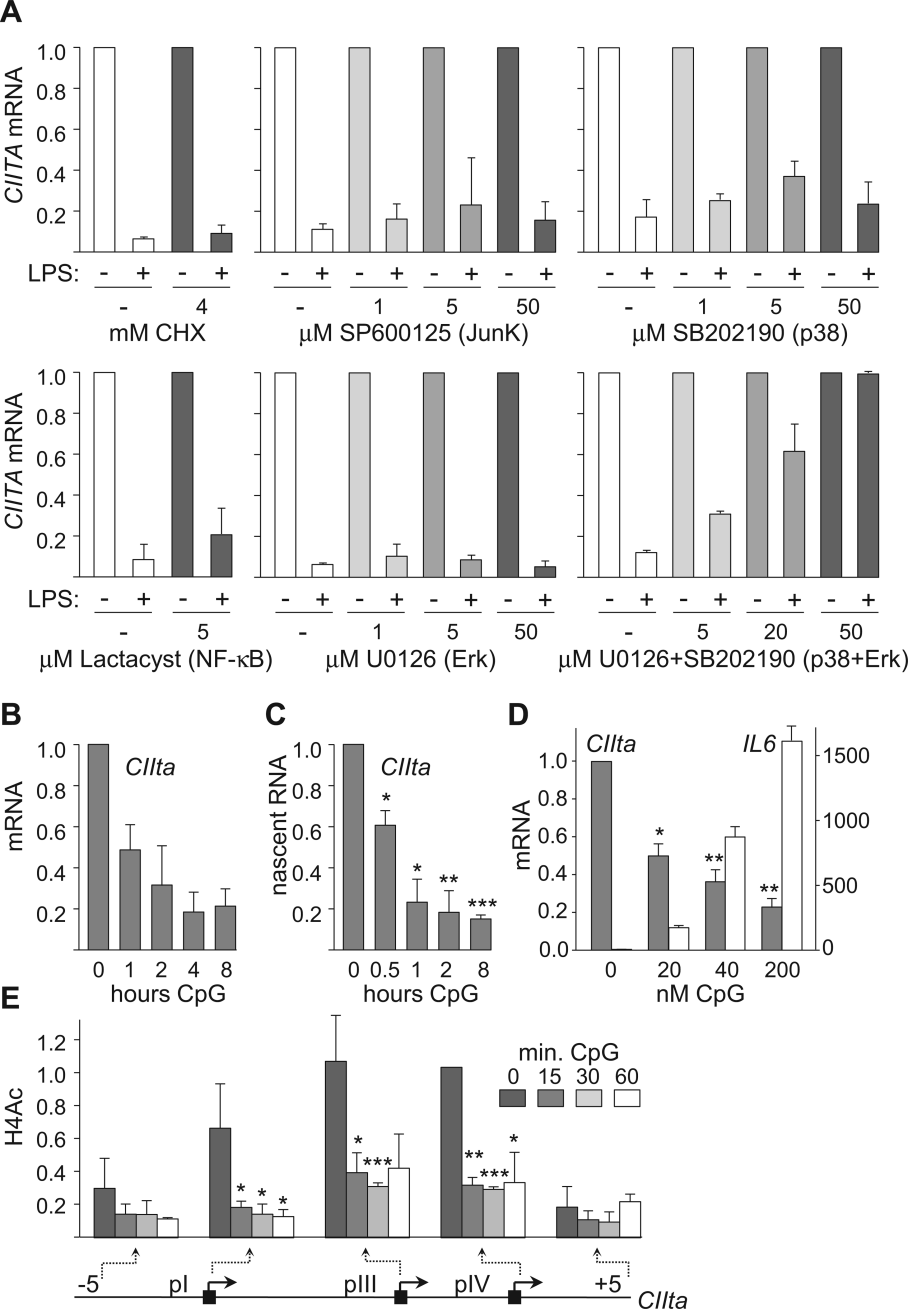
*CIITA* silencing is a conserved primary response mediated by the p38 and ERK pathways. (**A**) CIITA mRNA was quantified in immature and 4 h-LPS-treated Mo-DCs in the presence of the indicated concentrations of cycloheximide (CHX), Jun kinase inhibitor SP600125, p38 inhibitor SB202190, NF-κB inhibitor lactacystin, ERK inhibitor U0126 and U0126 + SB202190. Results are represented relative to immature DCs. Results are derived from two experiments. (**B**) CIITA mRNA was quantified in DC^2114^ cells exposed to CpG for the indicated times. Results are presented relative unstimulated DCs. Results are derived from two experiments. (**C**) Nascent CIITA RNA was quantified in DC^2114^ cells. exposed to CpG for the indicated times. Results are presented relative unstimulated DCs. Statistical significance was derived from three experiments: *, *P* < 0.05, **, *P* < 0.01, ***, *P* < 0.001. (**D**) CIITA and Il6 mRNAs were quantified in DC^2114^ cells treated for 6 h with the indicated concentrations of CpG. Statistical significance was derived from three experiments: *, *P* < 0.05, **, *P* < 0.01. (**E**) H4Ac was measured in DC^2114^ cells activated with CpG for the indicated times at the indicated positions of the *CIIta* gene. Results are expressed relative to H4Ac at promoter IV in immature DCs. Statistical significance was derived from three experiments: *, *P* < 0.05, **, *P* < 0.01, ***, *P* < 0.001. All measurements were performed in triplicate for each experiment.

The rapid kinetics of *CIITA* silencing suggested that it is a primary response triggered by pre-existing signal-transduction pathways. This was confirmed by the finding that it was not abrogated by the protein-synthesis inhibitor cycloheximide (Figure 2A). Selective inhibitors were used to identify signal-transduction pathways mediating *CIITA* silencing (Figure 2A). Efficacy of the inhibitors was controlled by qRT-PCR experiments examining the expression of genes induced via the targeted pathways and western-blot experiments examining phosphorylation of signaling intermediates (data not shown). Inhibitors of NF-κB activation, including lactacystine (Figure 2A) and MG-132 (data not shown) had no impact on *CIITA* silencing. Inhibitors of the c-Jun N-terminal kinase JNK (SP600125), p38 (SB202190) and extracellular signal-regulated kinases ERK (U0126) MAPK pathways also had no impact when added individually. However, *CIITA* silencing was completely abrogated by blocking both the p38 and ERK pathways (Figure 2A).

Key features of the CIITA-silencing process were investigated in the mouse DC^2114^ cell line (22,23) stimulated with CpG. Rapid maturation-induced silencing of the *CIIta* gene was evident at the mRNA (Figure 2B) and nascent transcript (Figure 2C) levels, sensitive to lower doses of CpG than required for optimal IL6 induction (Figure 2D), and associated with rapid histone-deacetylation within its regulatory region (Figure 2E, Supplementary Figure S1B). Silencing of *CIIta* in mouse BM-DCs was previously reported to involve the p38 and ERK pathways (28).

### *CIITA* silencing is representative of a global transcriptional remodeling response

**Figure 3.**
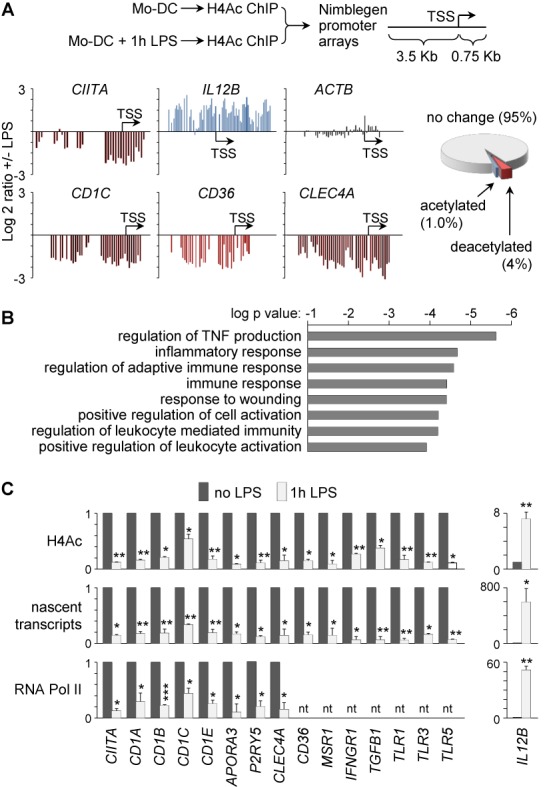
Identification of promoters undergoing H4-deacetylation upon Mo-DC maturation. (**A**) Schematic representation of the ChIP-chip strategy used to identify promoters that are deacetylated in Mo-DCs after 1 h of LPS treatment (top). Representative results for *CIITA*, *IL12B*, *ACTB*, *CD1C*, *CD36* and *CLEC4A* are provided: signal ratios between 1 h-LPS-treated and untreated Mo-DCs are represented on a log_2_ scale (bottom left). The percentages of promoters displaying increased or decreased H4Ac are shown (bottom right). (**B**) Gene-ontology analysis of genes exhibiting LPS-induced H4-deacetylation at their promoters was done using David (http://david.abcc.ncifcrf.gov/). (**C**) H4-deacetylation (top), nascent transcripts (middle) and pol-II occupancy (bottom) were quantified for the indicated genes in untreated and 1 h-LPS-treated Mo-DCs: results are expressed relative to untreated DCs; nt, not tested. Statistical significance was derived from three experiments: *, *P* < 0.05, **, *P* < 0.01, ***, *P* < 0.001. All measurements were performed in triplicate for each experiment.

To identify additional genes subjected to the same silencing process as *CIITA*, H4Ac-ChIP samples from immature Mo-DCs and 1 h-LPS-treated Mo-DCs were used to probe genomic arrays carrying a comprehensive set of human promoters (Figure 3A). ∼1000 promoters (∼4%) exhibited strong and reproducible H4-deacetylation (Figure 3A, Supplementary Figure S2A). Promoters exhibiting H4-deacetylation were more numerous than those displaying increased H4Ac (Figure 3A, ∼1%). The spatial distribution of deacetylated regions revealed a preference for positions close to the transcription-start-site (TSS, Supplementary Figure S2C). ChIP-chip experiments performed with our custom array confirmed that H4-deacetylation affected regions upstream of the TSSs of representative genes (Supplementary Figure S2B).

Gene-ontology analyses revealed that genes displaying promoter-deacetylation are significantly enriched in functions of high relevance for the immune system and DC biology (Figure 3B). For a selection of such functionally relevant genes, H4 and H3 deacetylation in 1 h-LPS-treated Mo-DCs was confirmed by qChIP experiments (Figure 3C and data not shown).

Key features of the silencing mechanism documented for *CIITA* were investigated for selected genes. Quantifications of nascent transcripts and Pol-II occupancy indicated that all tested genes exhibited rapid transcriptional downregulation and pol-II disengagement after 1 h of LPS exposure (Figure 3C). Downregulated genes exhibited a 2 to 20-fold reduction in transcription. LPS-induced transcriptional silencing preceded mRNA decay (Supplementary Figure S3A) and reduced protein expression (Supplementary Figure S3B) at representative genes. TLR ligands other than LPS also induced silencing (Supplementary Figure S2D). Finally, for selected genes, such as *IFNGR1*, it was confirmed that silencing is induced by low LPS concentrations (Supplementary Figure S3C) and blocked by combined inhibition of the ERK and p38 pathways (Supplementary Figure S3D).

### Rapid transcriptional silencing of deacetylated genes

**Figure 4.**
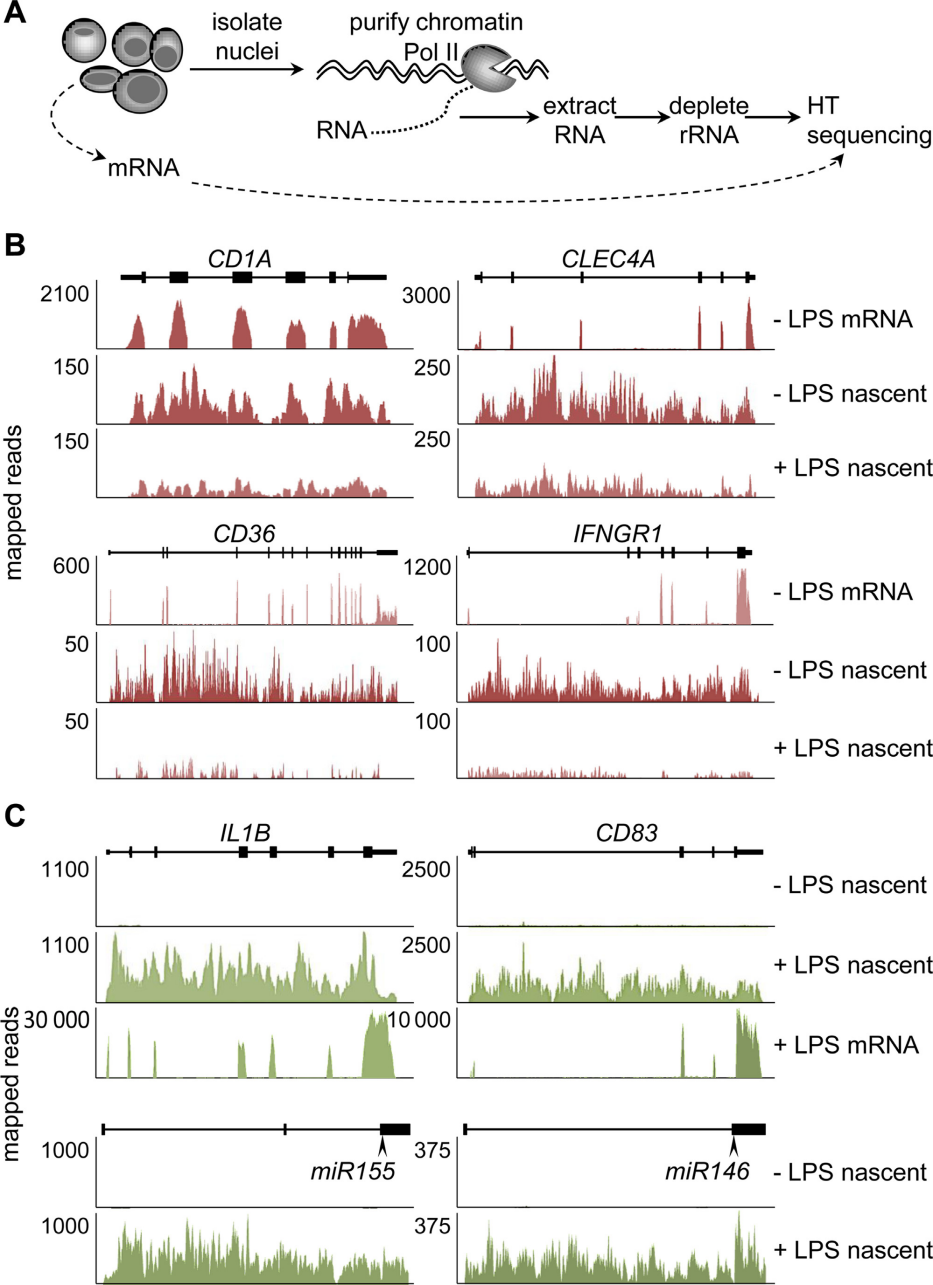
Transcriptome profiling by nascent-transcript sequencing. (**A**) Schematic representation of the strategy used for purifying and sequencing chromatin-bound nascent transcripts. mRNA was purified and sequenced in parallel. (**B** and **C**) Nascent-transcript-sequencing profiles are shown for representative silenced (B) and induced (C) genes: results are expressed as numbers of reads mapping to the genes in untreated and 1 h-LPS-treated Mo-DCs; schematic maps of the genes are depicted; exons are indicated as boxes; positions of mature microRNA sequences are indicated for microRNA genes; mRNA-sequencing profiles from untreated Mo-DCs are included as controls for protein-coding genes.

To assess global transcriptional consequences of histone-deacetylation, we explored the possibility of exploiting published microarray-based mRNA-expression data. A comparison of seven datasets for LPS-treated Mo-DCs (29–32) indicated that mRNA-profiling is not reliable for studying gene silencing. Reproducibility was significantly lower for silenced genes than induced genes: whereas most induced genes (75%) were reproduced in at least two experiments, most down-regulated genes (60%) were observed in only one experiment (Supplementary Figure S3E). Furthermore, key genes silenced in activated Mo-DCs, including *CIITA*, *MARCH1* and *CD1A* (Figure 1, Figure 3C and Supplementary Figure S3A and F) (20,21), were not found to be down-regulated in the microarray experiments, probably because of low expression-levels and/or mRNA half-life issues. We therefore developed a genome-wide approach for measuring global transcription rates, based on high-throughput sequencing of chromatin-bound primary transcripts (Figure 4A). Primary-transcript and mRNA preparations from immature Mo-DCs and 1 h-LPS-treated Mo-DCs were sequenced in parallel. Reproducibility between biological repeats was excellent as evidenced by examining global transcription profiles (Supplementary Figure S4A), spatial patterns of sequence-reads mapping to individual genes (Supplementary Figure S4B), and transcriptional changes observed for representative genes (Supplementary Figure S4C).

Three lines of evidence confirmed that the procedure quantifies primary transcripts. First, primary-transcript-reads mapped to the introns and exons of individual genes, whereas mRNA-reads mapped exclusively to exons (Figure 4B and C). Second, nascent-transcript-reads mapped to entire microRNA genes, not just mature-microRNA sequences (Figure 4C). Third, silencing was readily detectable for individual genes after 1 h of LPS treatment, well before decreased mRNA abundance was evident (Supplementary Figure S3A).

**Figure 5.**
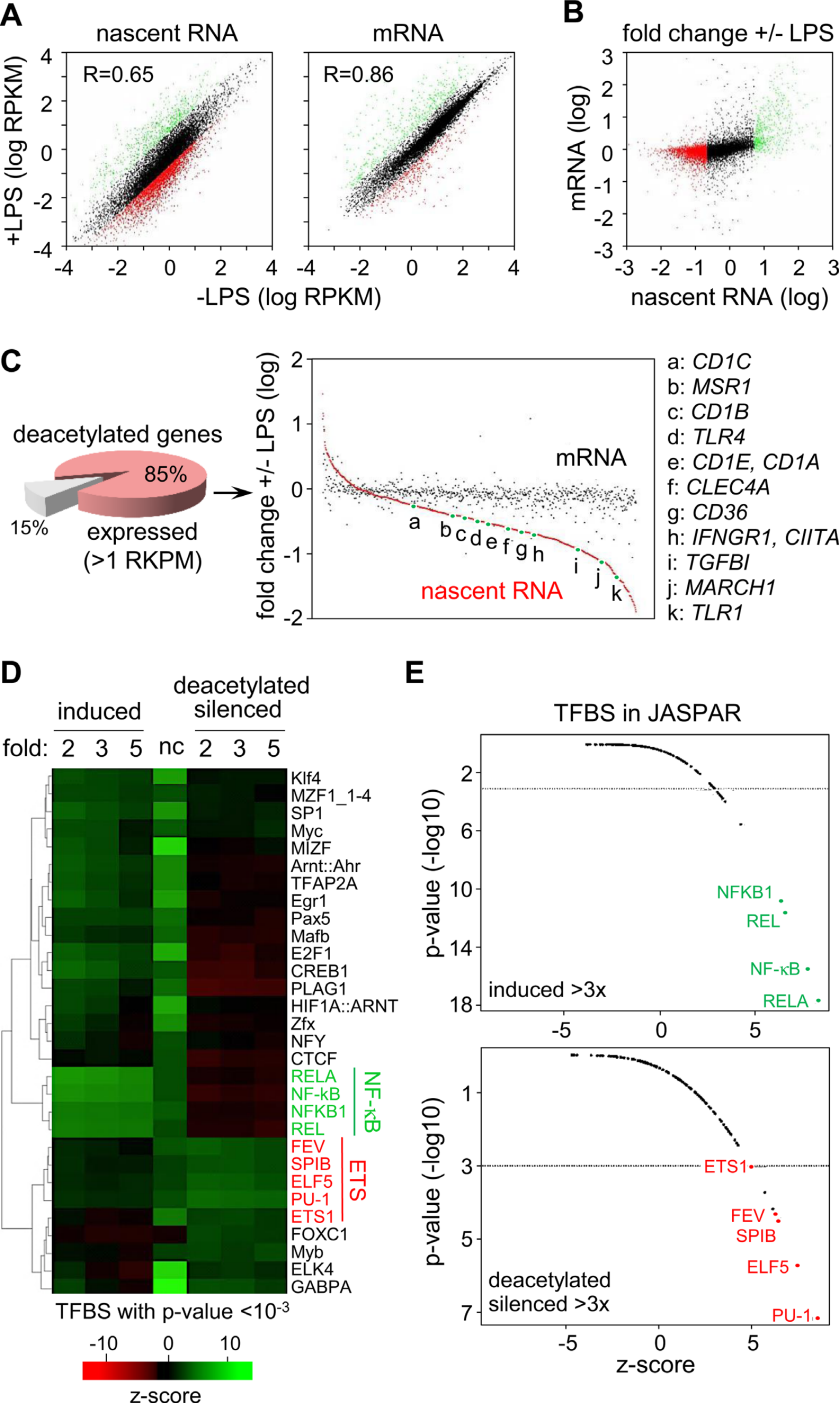
Characterization of nascent-transcript-sequencing data. (**A**) The dot plots show a global analysis of altered nascent-transcript (left) and mRNA (right) expression induced in 1 h-LPS-treated Mo-DCs: results are represented as RPKM (reads per kb per million) on a log scale; induced (>2x), silenced (>2x) and unchanged genes are represented as green, red and black dots, respectively. (**B**) The dot plot compares changes in nascent-transcript and mRNA expression induced in 1 h-LPS-treated Mo-DCs: results are represented as fold change on a log scale; genes that are induced (>2x), silenced (>2x) and unchanged at the nascent-RNA level are represented as green, red and black dots, respectively. (**C**) The pie chart shows the percentage of deacetylated genes expressed more than 1 RPKM in immature Mo-DCs. The dot plot shows 1 h-LPS-induced changes in nascent-transcript (red dots) and mRNA (black dots) expression for deacetylated genes; genes are ordered with respect to their change in nascent-transcript expression; positions of representative genes are highlighted. (**D**) TFBS enrichment analyses were performed for promoters of genes that are induced (>2, 3 or 5-fold), deacetylated and silenced (>2, 3 or 5-fold), or exhibit no change (nc) in expression in Mo-DCs after 1 h of LPS treatment. TFBSs were defined according to JASPAR. The heat map shows the relative enrichment (*z*-score) of TFBSs that are significantly over-represented (*P*-value < 10^−3^). (**E**) The graphs summarize the *z*-scores and *P*-values for all TFBSs in genes that are induced >3-fold (top) or deacetylated and silenced >3-fold (bottom): NF-κB (green) and ETS (red) TFBSs are highlighted.

Examinations of selected genes indicated that nascent-transcript sequencing allows reliable quantification of transcription rates at genes that are silenced or induced in 1 h-LPS-treated Mo-DCs, including protein-coding and microRNA genes (Figure 4B, C and Supplementary Figure S4C). Global analyses indicated that markedly more changes in expression were evident at the primary-transcript level than at that of mRNA-abundance (Figure 5A and Supplementary Table S1). Significantly more genes were down-regulated at the nascent transcript level than at the mRNA level (Figure 5B and Supplementary Table S1), suggesting that most reductions in transcription rate induced by 1 h of LPS stimulation do not yet have a major impact on mRNA abundance. These results demonstrate that assessing gene silencing by nascent-transcript sequencing is significantly more reliable and has strongly improved temporal resolution compared to mRNA-profiling.

Eight hundred forty-seven (85%) of the genes subjected to rapid histone-deacetylation were expressed in immature Mo-DCs above a baseline level of 1 read per kb per million (RPKM) (Figure 5C). 590 (70%) of these genes exhibited >2-fold reduction in transcription rate after 1 h of LPS treatment, including all genes for which silencing was validated by qRT-PCR (Figure 5C and Supplementary Table S1). Reductions in transcription ranged from 2 to nearly 100-fold (Figure 5C), indicating that the repressed genes comprise genes that are turned off and genes that are down-modulated. As inferred from the global analyses (Figure 5B), reductions in mRNA abundance were not yet evident for most deacetylated-silenced genes (Figure 5C).

### PU.1-occupied sites mark silenced genes

Insight into the silencing mechanism was gained by analyzing predicted TFBSs. TFBS-enrichment was investigated in genes that are induced, unchanged in their expression, or deacetylated and silenced (Figure 5D). Induced genes were characterized by a strong enrichment in NF-κB TFBSs (Figure 5D, E and Supplementary Table S2). In sharp contrast, TFBSs for ETS-family members were strongly enriched in deacetylated-silenced genes (Figure 5D, E and Supplementary Table S2). Both patterns of TFBS-enrichment differed markedly from that observed for genes that are unchanged in their expression (Figure 5D). The different patterns of TFBS-enrichment suggest that the three sets of genes are controlled by distinct regulatory mechanisms.

**Figure 6.**
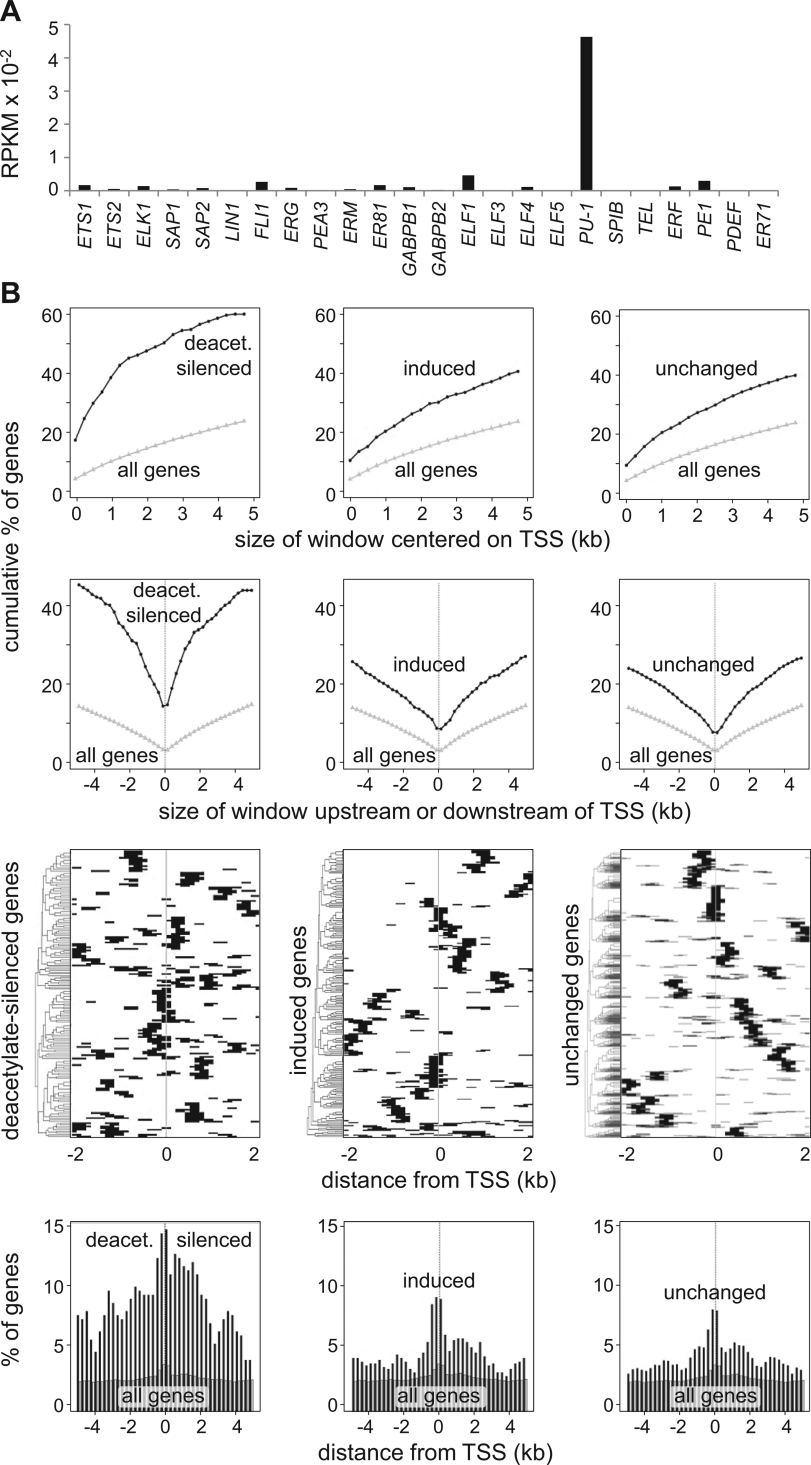
Mapping of PU.1-binding in Mo-DC. (**A**) Relative expression (mRNA-sequencing) of mRNAs encoding ETS family members in immature Mo-DCs. (**B**) PU.1-ocuppied sites (ChIP-sequencing) were analyzed in the promoter regions of genes that are deacetylated and silenced >5-fold (left column), induced >5-fold (center column), or exhibit no change in expression (right column) in Mo-DCs after 1 h of LPS treatment. Panels show the percent of genes having at least 1 PU.1 peak within a window of the indicated size centered on the TSS (first row), the percent of genes containing at least 1 PU.1 peak within the indicated distance upstream or downstream of the TSS (second row), heat maps indicating the positions of PU.1 peaks (black lines) within 4 kb regions centered on the TSS (third row) and the percent of genes having at least one PU.1 peak situated at the indicated distance upstream or downstream of the TSS (bottom row). In all graphs, the entire set of human genes was used as baseline reference. RPKM, reads per kb per million.

PU.1 is the most strongly expressed ETS-family member in Mo-DCs (Figure 6A and Supplementary Table S2). ChIP-sequencing experiments were performed to map PU.1 binding in Mo-DCs. The distribution of PU.1-bound sites was compared between the promoter regions of genes that are deacetylated-silenced, induced or unchanged in their expression in response to LPS (Figure 6B). The promoters of all human genes were used as baseline. PU.1-occupied sites were more frequent relative to baseline in all three subsets, distributed symmetrically upstream and downstream of the TSS, and enriched most strongly near the TSS. Surprisingly, PU.1-bound sites were most frequent in deacetylated-silenced genes. Binding was not affected by LPS treatment at most positions, indicating that silencing is not due to PU.1-disengagement (Supplementary Figure S5).

### Functions affected by epigenetic silencing

**Figure 7.**
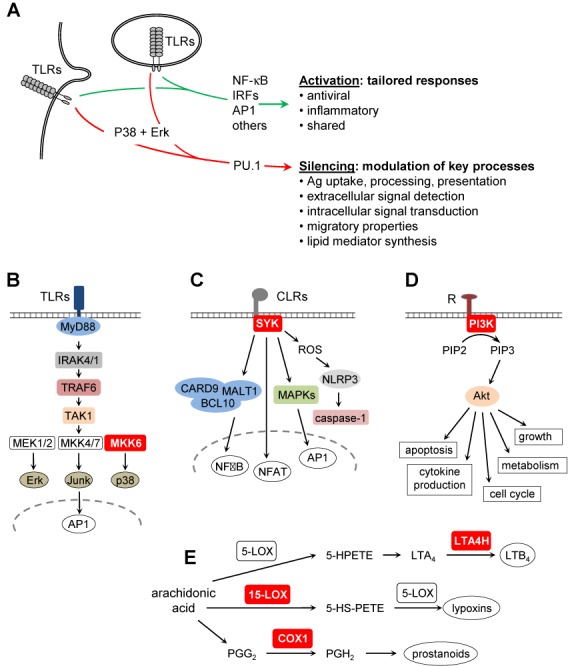
Functional relevance of rapid transcriptional silencing in activated DCs. (**A**) Schematic summary of signal-transduction pathways and functional consequences triggered by TLR-engagement in DCs: our results define a novel primary silencing pathway that is distinct from known gene induction mechanisms and modulates key processes during DC maturation. The silencing mechanism affects the expression of pivotal proteins (red boxes) implicated in: (**B**) TLR signaling (MKK6), (**C**) CLR signaling (SYK), (**D**) PI3K-Akt signaling (PI3K) and (**E**) icosanoid biosynthesis (LTA4H, 15-LOX, COX1).

Gene-ontology analyses demonstrated that genes subjected to silencing are strongly enriched in immune-system processes, including numerous genes implicated in DC function (Figure 3B and Supplementary Table S3). The most relevant processes include Ag uptake and presentation, extracellular-signal detection, signal transduction, lipid metabolism, cell migration and cytokine production (Figure 7 and Supplementary Table S3, see ‘Discussion’ section). Although down-regulated expression during DC-maturation was reported for certain genes, such as *CIITA*, *MARCH1* and *CD36* (20,21), for the majority this has not been documented. Rapid epigenetic silencing is thus a newly identified mechanism that concerns numerous functionally relevant genes and makes a major contribution to transcriptional reprogramming of DCs at an early stage of the maturation process.

## DISCUSSION

Analysis of *CIITA* silencing led to the identification of a novel silencing mechanism that makes a substantial contribution to reshaping of the transcription program during DC maturation. This mechanism is conserved between humans and mice, triggered by multiple maturation stimuli, dependent on combined activation of the p38 and ERK MAPK signal-transduction pathways, associated with extensive histone-deacetylation over large regulatory domains, and leads to transcriptional silencing of hundreds of genes, many of which have functions of central relevance for DC biology. The extent of repression is variable, ranging from genes that are moderately down-regulated to genes that are essentially turned off.

Several methods for analyzing gene-expression profiles directly at the level of transcription have been reported (7,33,34). We developed a simple and robust procedure similar to that recently reported elsewhere (7). This method relies on high-throughput sequencing of chromatin-bound primary transcripts. It proved to be highly reliable for measuring rapid changes in transcription-rates with a high degree of sensitivity and temporal resolution. It is particularly valuable for dissecting dynamic transcriptional silencing mechanisms, which is difficult to achieve by mRNA quantification, particularly for mRNAs having long half-lives. Furthermore, identifying sets of genes that are repressed in a temporally coordinated manner cannot be achieved reliably by mRNA-expression profiling because distinct mRNAs can have substantially different stabilities.

The silencing mechanism described here constitutes a primary response that is independent of *de novo* protein synthesis and essentially complete within 1 h. These features distinguish it from repression mechanisms operating during later stages of DC maturation, such as the dampening or termination of gene expression by the class II TFs ATF3, BCL6 and PRDM1 (17–19). It also precedes the establishment of epigenetic modifications that repress inducible cytokine-gene expression at late stages of myeloid cell activation, such as those implicated in conferring endotoxin tolerance in activated macrophages (35).

Promoters of induced genes are characterized by an increased frequency of NF-κB TFBSs. In contrast, genes subjected to silencing exhibit an under-representation of NF-κB TFBSs and a high frequency of TFBSs for ETS-family members. The under-representation of NF-κB-binding sites is consistent with the observation that silencing is independent of NF-κB activation. However, an increased frequency of ETS-family TFBSs in silenced genes was unexpected.

ChIP-seq experiments confirmed that PU.1-bound sites are most frequent in the promoters of silenced genes. In mice, Pu.1 is a lineage-specification TF playing critical roles in hematopoiesis, including the development of myeloid and lymphoid cells (36,37). Conditional gene-deletion demonstrated that Pu.1 is required for the development of all DC subsets (38). Genome-wide binding studies performed in various cell types, including DCs, have suggested that Pu.1 functions as a ‘pioneer’ factor that establishes a permissive chromatin landscape at enhancers controlled by lineage-specific and inducible TFs (14,15). Given this function, the finding that PU.1-bound sites are more frequent in promoters subjected to inducible repression than in inducible or constitutive promoters was unanticipated and suggests a novel role for this TF, namely that it marks genes poised for inducible silencing. This is consistent with reports implicating PU.1 in the repression of specific genes (39). It has notably been suggested to repress promoter I of *CIITA* in mouse and human DCs (19). Repression by PU.1 has been attributed to its ability to recruit HDACs (40,41), a mechanism consistent with the finding that histone-deacetylation is the primary event associated with the silencing process we have unveiled. However, we have been unable to demonstrate selective recruitment of specific HDACs to deacetylated-silenced promoters during Mo-DC maturation (data not shown).

The silencing mechanism is anticipated to have profound impacts on Ag capture, processing and presentation. First, the silencing process leads to increased cell-surface MHCII expression and reduced *de novo* MHCII synthesis. Enhanced cell-surface MHCII expression results from down-regulation of the E3 ubiquitin ligase MARCH1, which increases MHCII turnover by promoting their ubiquitination and degradation (20,42). Decreased MHCII-synthesis is due to abrogated CIITA expression (21). Concomitant silencing of the *MARCH1* and *CIITA* genes ensures that MHCII-restricted Ag presentation focuses on Ags captured prior to DC-activation. Second, reduced expression of the endocytic adaptors Epsin-1 and Epsin-2 is expected to hinder clathrin-mediated endocytosis (43) and could contribute to impaired Ag-uptake by mature DCs (44). Third, silencing of genes encoding endocytic CLRs, such as *CLEC10A* (encoding MGL, macrophage galactose C-type lectin receptor), *CLEC4A* (encoding DCIR, DC immune-receptor) and *CLEC12A* (encoding MICL, myeloid inhibitory C-type lectin) is likely to impair Ag-uptake in mature DCs. These CLRs bind glycans displayed on pathogen or self-derived proteins, mediating their internalization and delivery to Ag-loading compartments, and promoting Ag-presentation to CD4^+^ T cells and cross-presentation to CD8^+^ T cells (45). Fourth, down-regulation of the scavenger receptor CD36 will impair phagocytosis of pathogens and apoptotic cells (46,47). Fifth, silencing of the gene encoding insulin-regulated-aminopeptidase (IRAP) is likely to contribute to impaired cross-presentation by mature DCs (48). IRAP is an endosomal protease required for cross-presentation because it generates peptides suitable for MHCI-mediated Ag presentation by catalyzing amino-terminal trimming of peptides derived from internalized proteins (48). Sixth, down-regulation of CD1A, CD1B, CD1C and CD1E expression is anticipated to lead to reduced *de novo* presentation of lipid and glycolipid Ags, in a manner analogous to reduced *de novo* MHCII synthesis. Finally, silencing of BTN3A1 expression will reduce presentation of phosphorylated Ags. BTN3A1 was recently demonstrated to present phosphorylated Ags to Vγ9Vδ2 T cells (49). In summary, the silencing mechanism described here has a broad impact on Ag capture, processing, presentation and cross-presentation, including effects on the presentation of peptide, lipid and phosphorylated Ags.

A second major function affected by the silencing mechanism is the ability to detect and respond to extracellular signals. First, silencing of the *CCR1* and *CCR5* genes contributes to the chemokine receptor switch (induction of CCR7, down-regulation of CCR1 and CCR5) allowing mature DCs to exit peripheral tissues and home to lymphoid tissues (50). Second, down-regulation of TLR1, 3, 4 and 5 is anticipated to reduce responsiveness to pathogens and endogenous danger signals. Third, reduced DCIR, MICL and MGL expression will reduce signaling induced by these endocytic CLRs. The cytoplasmic tails of DCIR and MICL contain ITIM motifs, which recruit the SHP-1 and SHP-2 phosphatases, thereby inhibiting TLR and CLR induced DC-maturation and inflammatory-cytokine expression (45,51). Mgl engagement also exerts an anti-inflammatory role in mouse models (45,51). Silencing of DCIR, MICL and MGL may thus remove an inhibitory influence on DC maturation and function. Fourth, genes encoding pivotal components of three major signal-transduction pathways are silenced. Spleen tyrosine kinase (SYK) plays a central role in signaling induced by the engagement of many CLRs, such as Dectin-1, Dectin-2 and Mincle, as well as in TLR4-induced signaling (52). MAP-kinase-kinase 6 (MAP2K6) plays a crucial role in the p38 MAPK cascade, which is activated by TLR-engagement and required for DC maturation (29). The p110α (PIK3CA) and p110γ (PIK3CG) catalytic subunits of class I phosphoinositide-3-kinases play pivotal roles in diverse receptor-induced signal-transduction pathways (53). Collectively, the repression of genes encoding diverse ligand-activated receptors and pivotal intracellular signaling molecules implies that the silencing process we have defined induces profound remodeling of the responsiveness of DCs to environmental signals.

A third process affected by the silencing mechanism is lipid mediator production. Reduced prostaglandin-endoperoxidase-synthesase-1 (PTGS1) expression will impair the production of prostanoids. Decreased leukotriene-A4-hydrolase (LTA4H) expression will impair leukotriene-B4 (LTB_4_) synthesis. Silencing of arachidonate-15-lipoxygenase (ALOX15) expression will reduce the synthesis of several bioactive lipids, including lipoxins. Reduced expression of these enzymes should have a dramatic impact on lipid-mediator synthesis. The functional consequences of this alteration are difficult to predict, as lipid-biosynthesis pathways are complex and lipid mediators can exert distinct pro-inflammatory and immunosuppressive effects on diverse cell types. Knowledge on the patterns of lipid-mediator production by DCs remains fragmentary. Our results suggest that this aspect of DC function has been neglected and requires investigation.

In conclusion, genes subjected to the silencing mechanism we have characterized are implicated in key biological processes. These notably include Ag uptake, processing, presentation and cross-presentation, extracellular-signal detection, intracellular signal-transduction and lipid-mediator synthesis. Rapid maturation induced silencing thus makes a major contribution to the remodeling of DC function in response to activation signals.

## Accession Numbers

Complete data sets are available at the Gene Expression Omnibus (accession numbers GSE58864, GSE58961).

## SUPPLEMENTARY DATA

Supplementary Data are available at NAR Online.

SUPPLEMENTARY DATA
