# Supplementary material for: Extensive remodeling of DC function by rapid maturation-induced transcriptional silencing
Source: Nucleic Acids Res. 2014 Aug 7;42(15):9641–55. doi: 10.1093/nar/gku674 (PMC4150779; doi:10.1093/nar/gku674)
Supplement: SUPPLEMENTARY DATA [file supp_42_15_9641__index.html]

Extensive remodeling of DC function by rapid maturation-induced transcriptional silencing — Extensive remodeling of DC function by rapid maturation-induced transcriptional silencing — SUPPLEMENTARY DATA 

# Extensive remodeling of DC function by rapid maturation-induced transcriptional silencing

## SUPPLEMENTARY DATA

**Files in this Data Supplement:**

- SUPPLEMENTARY DATA
