# Supplementary material for: Extensive remodeling of DC function by rapid maturation-induced transcriptional silencing
Source: Nucleic Acids Res. 2014 Aug 7;42(15):9641–55. doi: 10.1093/nar/gku674 (PMC4150779; doi:10.1093/nar/gku674)
Supplement: SUPPLEMENTARY DATA [file supp_gku674_nar-01420-x-2014-File009.pdf]

Supplementary Figures and Tables

Figure S1. Histone deacetylation induced at the *CIITA/CIIta* gene during DC maturation. (**A**) H3Ac (top) and H3K4trim (bottom) were measured in Mo-DCs activated with LPS for the indicated times at the indicated positions of the *CIITA* gene. Results are expressed relative to promoter IV in immature DCs. Results are derived from 2 experiments. (**B**) H3Ac was measured by qChIP in mouse DC[2114] cells stimulated with CpG for the indicated times. Results are expressed relative to promoter IV in unstimulated cells. Statistical significance was derived from 3 experiments: *, $p<0.05$. All measurements were performed in triplicate for each experiment.

Figure S2. Identification of promoters undergoing H4-deacetylation upon Mo-DC maturation. (**A**) Results derived from three independent experiments are shown for representative promoters displaying a reduction in H4Ac (*CD1A*, *TGFBI*, *P2RY)*, no change in H4Ac (*EEAF1*) or an increase in H4Ac (*IL12B*). Results are represented as signal ratios between 1h-LPS-treated and untreated Mo-DCs (top rows), and as deacetylation peaks calculated by NimbleScan software (bottom rows). (**B**) H4Ac-profiling at the *BTN3A1* and *BAT1* genes was performed using our custom Nimblegen microarray. H4Ac in untreated Mo-DCs (blue) was determined as the signal ratio between immature DCs (iDC) and input DNA. H4-deacetylation (red) was determined as the signal ratio between iDCs and DCs exposed to LPS for 1h. Signal ratios are represented on a log 2 scale. TSS positions are shown below. (**C**) The bar graph represents the overall distribution of H4-deacetylation peaks relative to the TSS of all deacetylated promoters. (**D**) CIITA, CD1C, ADORA3, P2RY14, TLR5 and P2RY5 mRNA levels were quantified in Mo-DC exposed for 24h to the indicated stimuli. Results are expressed relative to unstimulated Mo-DCs.

Figure S3. Characterization of representative silenced genes. (**A**) mRNAs for the indicated genes were quantified (qRT-PCR) in Mo-DCs exposed to LPS for the indicated times: results are expressed relative to untreated cells. (**B**) Western blotting was performed to quantify the indicated proteins in Mo-DC exposed to LPS for the indicated times. (**C**) IFNGR1 and IL6 mRNAs were quantified (qRT-PCR) in Mo-DC treated for 6h with the indicated concentrations of LPS: results are expressed relative to untreated DCs for IFNGR1 mRNA, and DCs treated for 6h with the maximum dose of LPS for IL6 mRNA. (**D**) IFNGR1 and CIITA mRNAs were quantified in Mo-DCs treated with LPS for the indicated times in the absence or presence of the indicated concentrations of U0126+SB202190: results are expressed relative to immature Mo-DCs. (**E**) The pie charts depict the reproducibility of transcriptional changes observed in 7 published microarray data sets. The results represent the distribution of up-regulated (left) and down-regulated (right) mRNAs observed in 1, 2, 3, 4, 5, 6, or 7 of the data sets. Reproducibility is markedly better for up-regulated mRNAs than for down-regulated mRNAs. (**F**) mRNA (top) and nascent transcript (bottom) expression were measured (qRT-PCR) for *CIITA*, *MARCH1* and *CD1A* genes in Mo-DC treated with LPS for the indicated times. Silencing at the level of transcription is almost complete within 1h, before a significant reduction in mRNA is observed.

Figure S4. Reproducibility of nascent-transcript sequencing. (**A**) Comparison of results derived from two independent nascent-transcript (left) and mRNA (right) sequencing experiments performed for immature Mo-DCs and Mo-DCs treated for 1h with LPS.

(**B**) Nascent-transcript-sequencing profiles from 3 independent experiments are shown for two representative genes (*CD1C* and *CLEC4A*): results are expressed as numbers of reads mapping to the genes in immature Mo-DCs; schematic maps of the genes are depicted; exons are indicated as boxes. (**C**) Quantifications obtained in 3 independent nascent-transcript-sequencing experiments are shown for representative genes that are silenced (*CIITA*, *CD1A*, *CD1B*, *CD1C*, *CD1E*, *CLEC4A*, *IFNGR1* and *TGFBI*) or induced (*TNF*, *IL1B* and *IL6*) by treatment of Mo-DCs with LPS for 1h: results are represented as RPKM (reads per Kb per million); the fold repression is indicated for the silenced genes.

Figure S5. Reproducibility of PU.1-binding sites mapped by ChIP-sequencing in Mo-DCs. PU.1-binding peaks obtained in two independent experiments performed with immature Mo-DCs (iDC) and Mo-DCs stimulated for 1h with LPS are shown for representative deacetylated-silenced (*CIITA, ERP29*, *KCNK6*, *MS4A6A*) and induced (*CCL1*) genes. Red and blue profiles represent sequence reads mapping to the two strands of the DNA.

Table S1. RNA-seq analysis of induced and repressed genes.

Table S2. TFBS-enrichment in promoters of genes that are induced (*Table S1A*) or deacetylated and silenced (*Table S1B*) in Mo-DCs treated for 1h with LPS.

Table S3. Functions and genes affected by silencing.

Figure S1

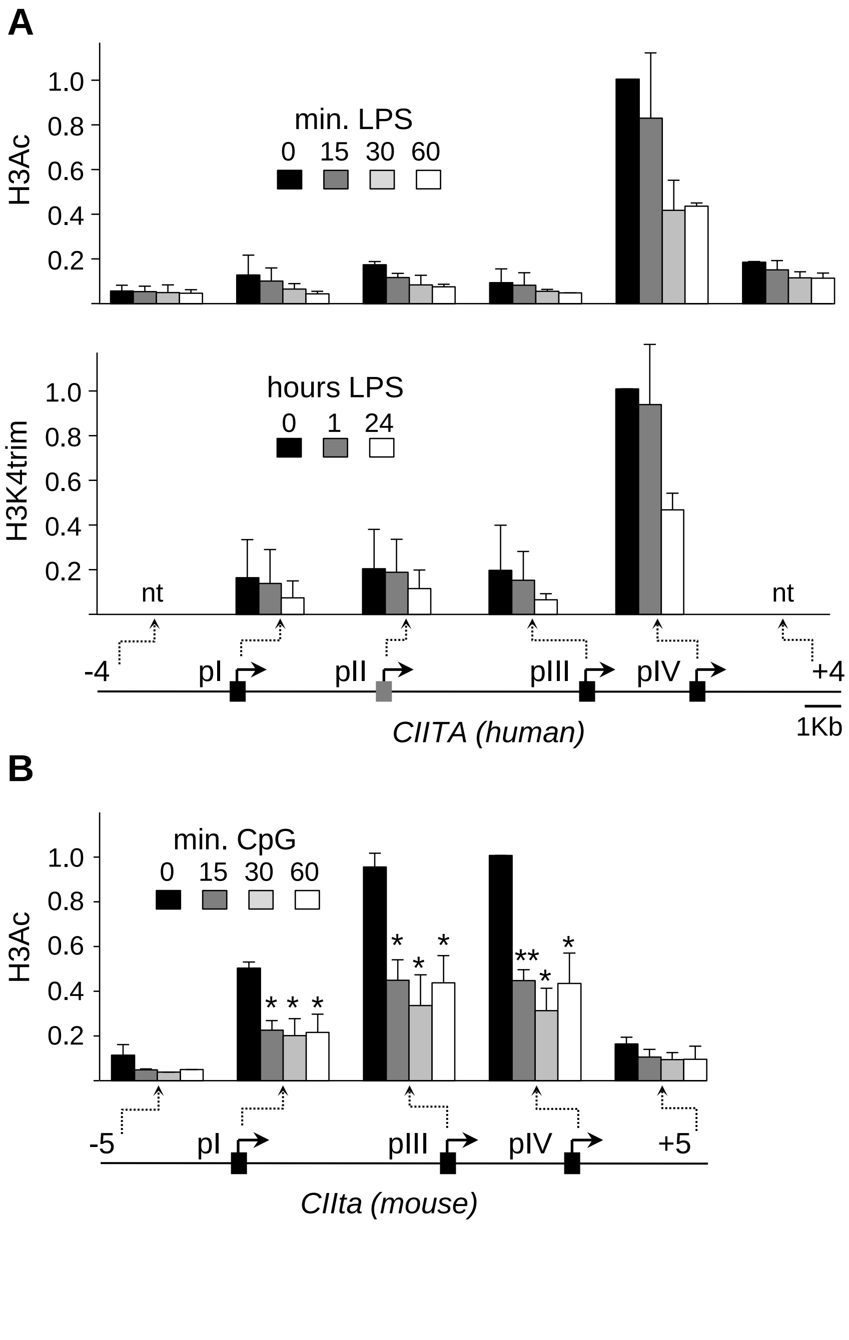
A
H3Ac
min. LPS
0 15 30 60
H3K4trim
hours LPS
0 1 24
nt
nt
-4
pI
pII
pIII
pIV
+4
1Kb
CIITA (human)
B
H3Ac
min. CpG
0 15 30 60
-5
pI
pIII
pIV
+5
CIIta (mouse)

Figure S2

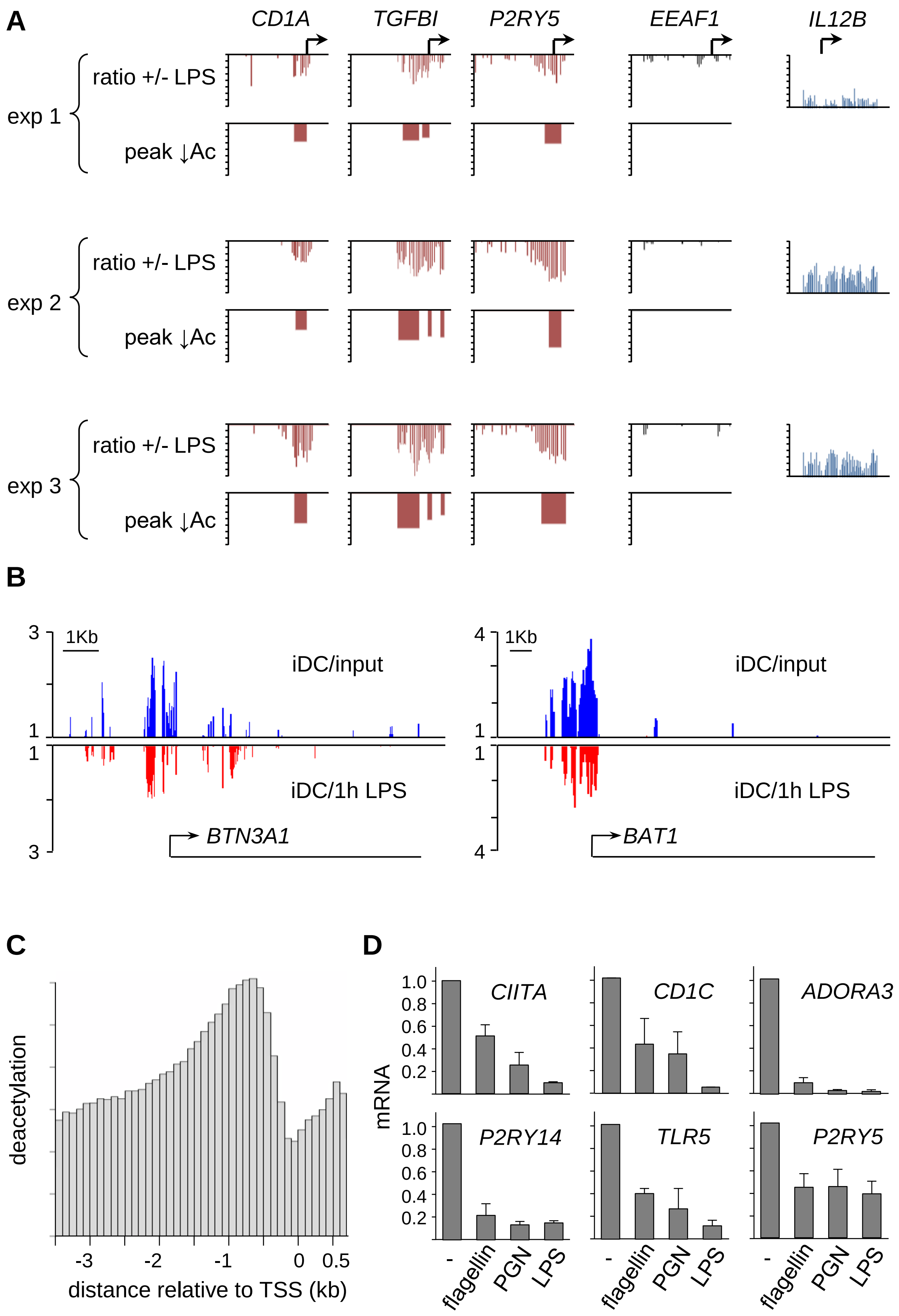

A
CD1A
TGFBI
P2RY5
EEAF1
IL12B
exp 1
exp 2
exp 3
ratio +/- LPS
peak ↓Ac
B
1Kb
iDC/input
iDC/1h LPS
BTN3A1
BAT1
3
1
4
C
deacetylation
distance relative to TSS (kb)
-3
-2
-1
0
0.5
D
mRNA
CIITA
CD1C
ADORA3
P2RY14
TLR5
P2RY5
1.0
0.8
0.6
0.4
0.2
-
flagellin
PGN
LPS

Figure S3

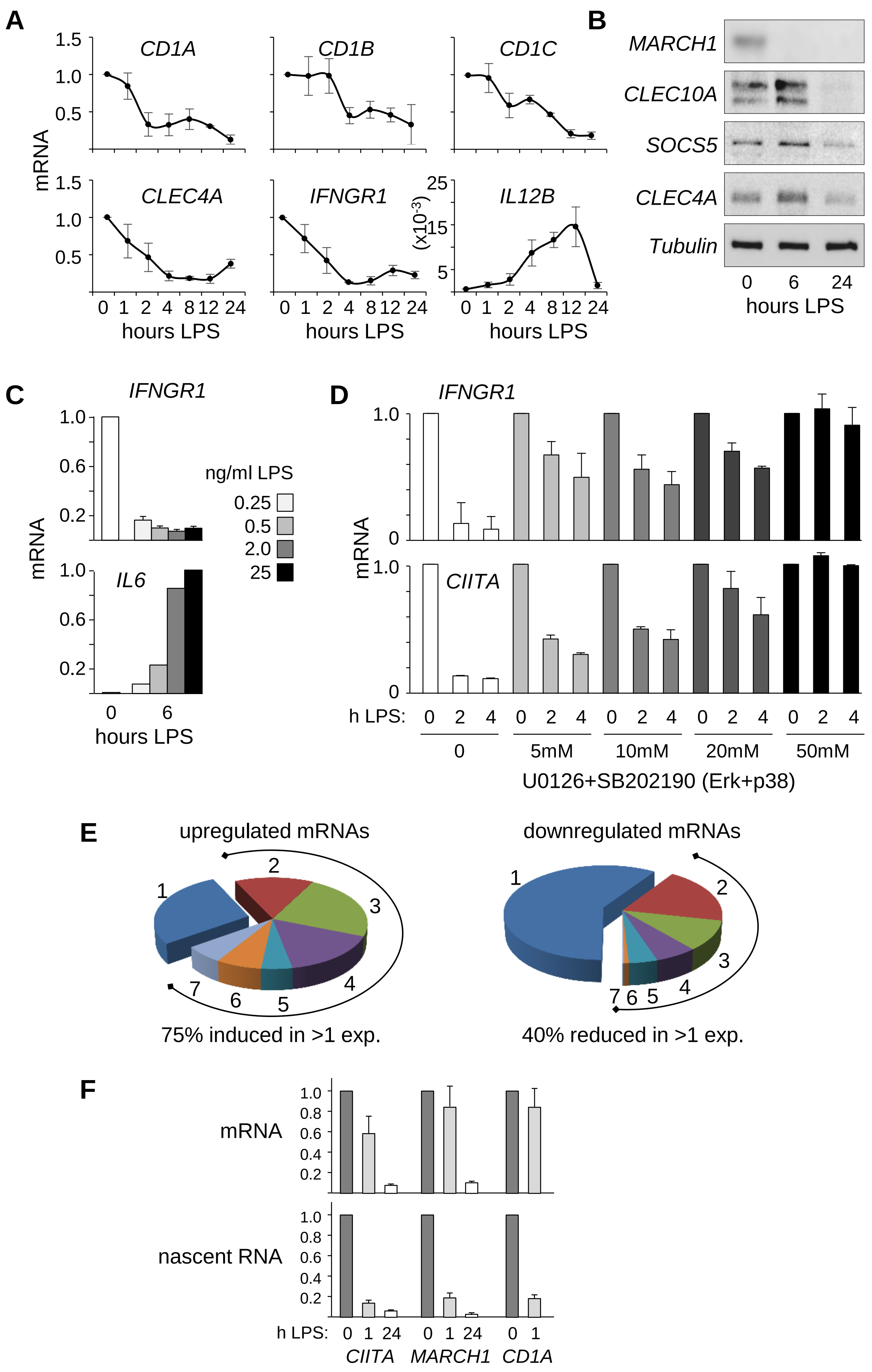

A
mRNA
CD1A
CD1B
CD1C
CLEC4A
IFNGR1
IL12B
1.5
1.0
0.5
25
15
5
(x10-3)
0 1 2 4 8 12 24
hours LPS
B
MARCH1
CLEC10A
SOCS5
CLEC4A
Tubulin
0 6 24
hours LPS
C
IFNGR1
IL6
mRNA
1.0
0.6
0.2
ng/ml LPS
0.25
0.5
2.0
25
0 6
hours LPS
D
IFNGR1
CIITA
mRNA
1.0
0
h LPS: 0 2 4 0 2 4 0 2 4 0 2 4 0 2 4
0
5mM
10mM
20mM
50mM
U0126+SB202190 (Erk+p38)
E
upregulated mRNAs
downregulated mRNAs
1 2 3 4 5 6 7
75% induced in >1 exp.
40% reduced in >1 exp.
F
mRNA
nascent RNA
1.0
0.8
0.6
0.4
0.2
h LPS: 0 1 24 0 1 24 0 1
CIITA
MARCH1
CD1A

Figure S4

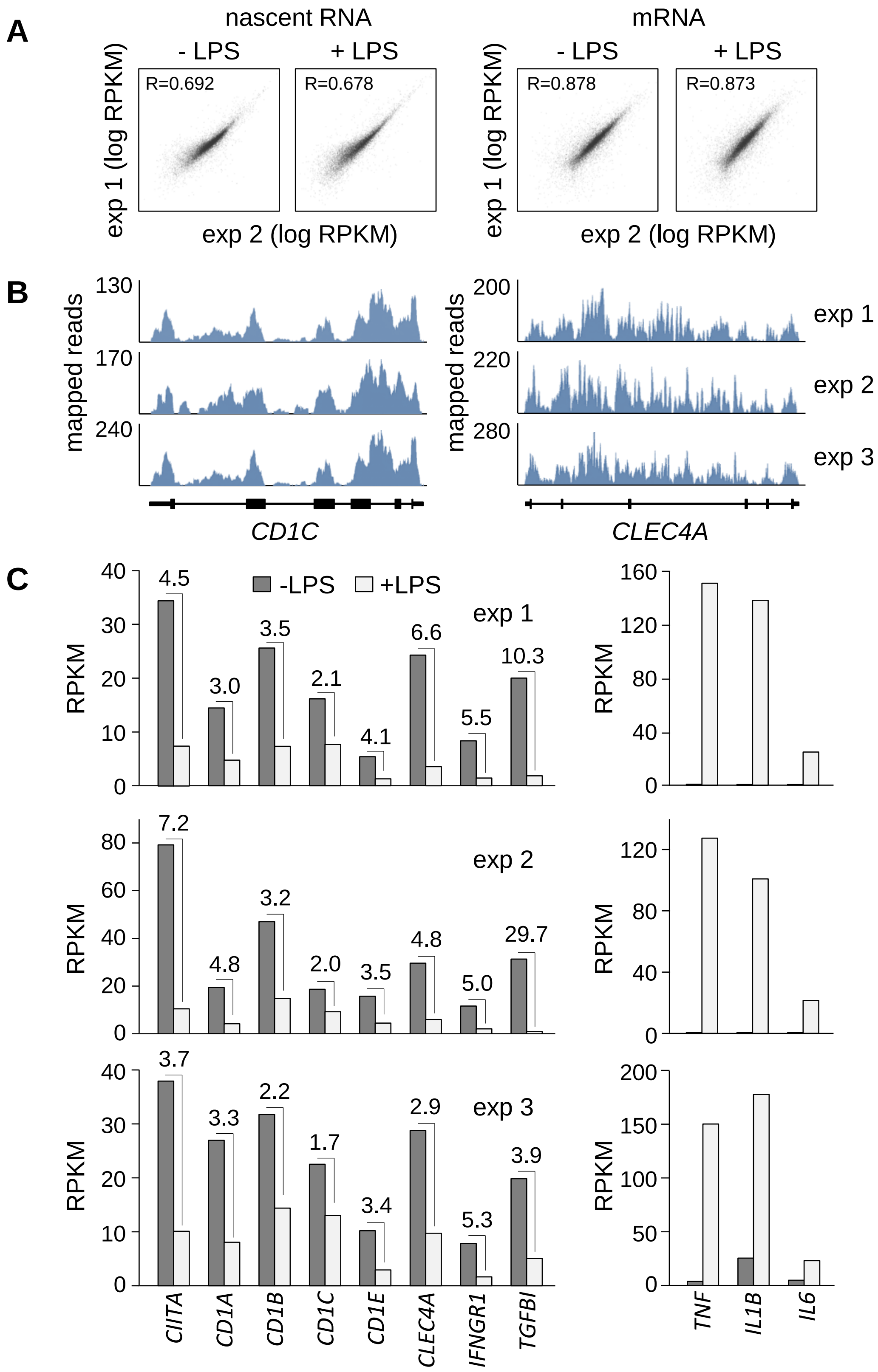
A
nascent RNA
mRNA
- LPS
+ LPS
R=0.692
R=0.678
R=0.878
R=0.873
exp 1 (log RPKM)
exp 2 (log RPKM)
B
mapped reads
130
170
240
200
220
280
exp 1
exp 2
exp 3
CD1C
CLEC4A
C
-LPS
+LPS
RPKM
exp 1
exp 2
exp 3
4.5
3.0
3.5
2.1
4.1
6.6
5.5
10.3
7.2
4.8
3.2
2.0
3.5
4.8
5.0
29.7
3.7
3.3
2.2
1.7
3.4
2.9
5.3
3.9
CIITA
CD1A
CD1B
CD1C
CD1E
CLEC4A
IFNGR1
TGFBI
TNF
IL1B
IL6

Figure S5

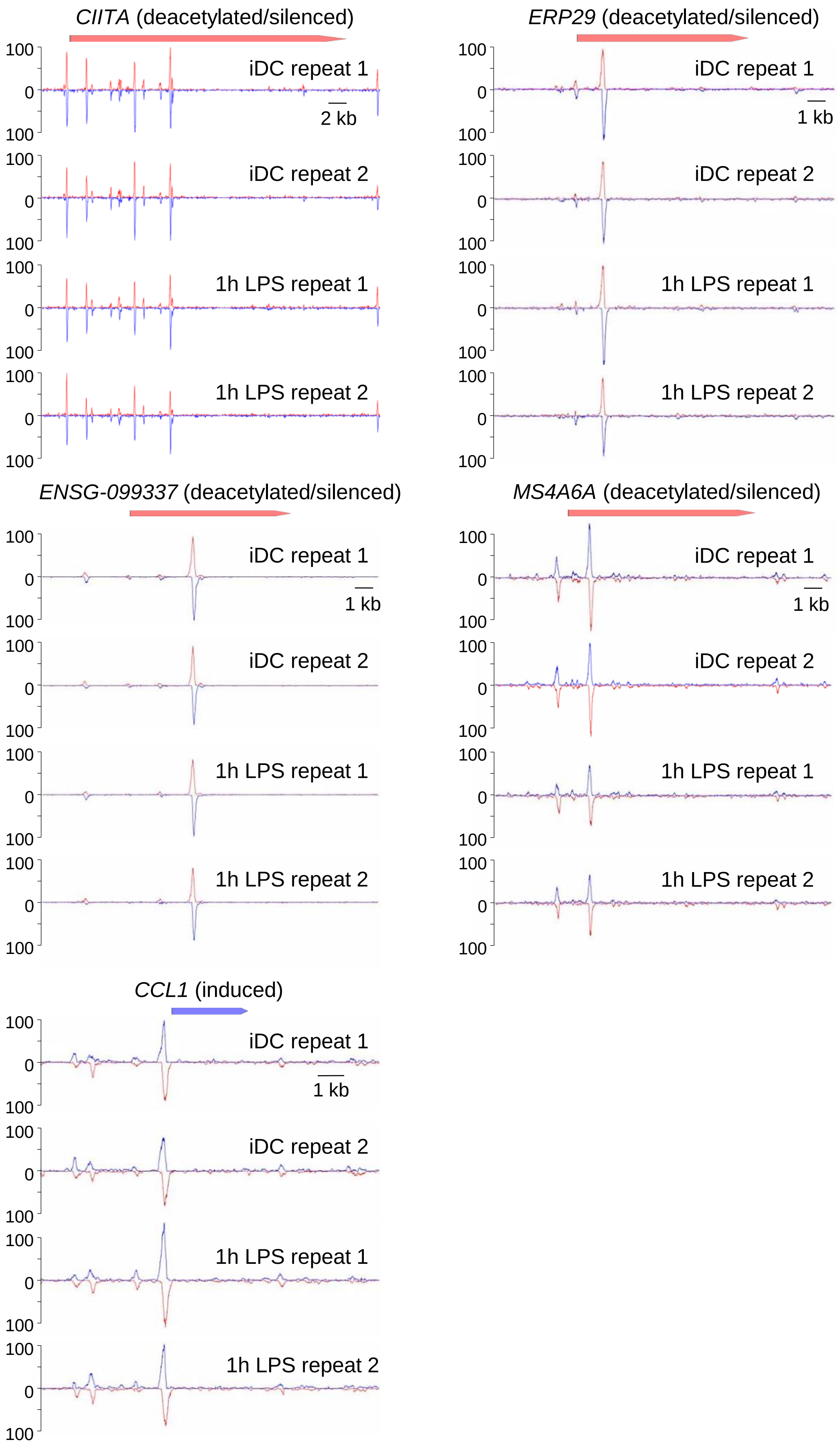
CIITA (deacetylated/silenced)
ERP29 (deacetylated/silenced)
ENSG-099337 (deacetylated/silenced)
MS4A6A (deacetylated/silenced)
CCL1 (induced)
iDC repeat 1
iDC repeat 2
1h LPS repeat 1
1h LPS repeat 2
2 kb
1 kb
100
0
100

Supplementary Table S1: RNA-seq analysis of induced and repressed genes

A. Genes[1] induced or down-regulated at the nascent transcript or mRNA levels.

	nascent transcripts		mRNA	
fold change	induced genes	repressed genes	induced genes	repressed genes
>2	2616	12746	1865	3041
>3	2013	9972	1508	1795
>5	1549	7050	1256	1251

[1] 22837 genes with at least one RPKM value >1.

B. Deacetylated genes[1] induced or down-regulated at the nascent transcript level.

	induced genes			silenced genes		
fold change	number	%	p-value[2]	number	%	p-value[2]
>2	32	3.8	ns	590	69.7	3.25e-16
>3	19	2.2	ns	464	54.8	1.18e-10
>5	9	1.1	ns	314	37.1	9.26e-05

[1] 847 deacetylated genes with at least one RPKM value >1.
[2] Enrichment in deacetylated gene group relative to all genes; Fisher-test; ns (not significant)

Supplementary Table S2. TFBS enrichment in induced and deacetylated-silence genes

A. Significantly enriched[1] TFBS in promoters of induced genes

	genes induced after 1h LPS						genes deacetylated-silenced after 1h LPS						[2]RPKM
	2 fold		3 fold		5 fold		2 fold		3 fold		5 fold		Mo-DC
TFBS	z	p	z	p	z	p	z	p	z	p	z	p	mRNA
Arnt::Ahr	4.4	2.1e-06	3.4	0.00021	2.2	0.01098	-0.4	0.68223	-0.7	0.76493	-0.9	0.82761	7.7
CREB1	5.3	1.2e-08	4.3	3.7e-06	2.4	0.00729	-2.3	0.99103	-2.4	0.99417	-1.1	0.87781	8
E2F1	4.6	6.7e-07	3.3	0.00028	2.6	0.00377	-1.4	0.92595	-2.0	0.98031	-0.8	0.79200	<1
Egr1	4.9	2.0e-07	3.3	0.00029	1.5	0.05383	-0.8	0.79656	-0.2	0.59870	-0.2	0.59016	3
HIF1A::ARNT	3.1	0.00063	2.2	0.01020	0.02	0.49090	0.2	0.38325	0.03	0.48414	-0.1	0.54043	53::14
Klf4	3.6	8.2e-05	2.9	0.00116	2.5	0.00439	0.5	0.27990	0.8	0.19229	0.8	0.19171	35
MIZF	4.1	1.0e-05	3.1	0.00057	1.1	0.12412	-0.06	0.52653	-0.2	0.61707	0.5	0.30244	7
Myc	3.5	0.00015	3.2	0.00045	0.8	0.18722	0.6	0.24846	0.6	0.23993	1.5	0.05514	30
MZF1_1-4	3.0	0.00076	3.0	0.00105	2.3	0.00826	1.1	0.12302	0.7	0.21062	0.04	0.48311	16
NF-kappaB	8.2	5.0e-18	7.8	5.2e-16	7.3	3.7e-14	-1.9	0.97587	-1.2	0.90119	-1.7	0.96145	17
NFKB1	6.7	1.9e-12	6.4	2.2e-11	6.1	1.9e-10	-0.8	0.79771	-0.6	0.75912	-1.0	0.85482	20
PLAG1	3.1	0.00052	2.7	0.00228	0.8	0.18224	-3.0	0.99907	-2.6	0.99640	-2.4	0.99356	<1
REL	7.0	1.2e-13	6.6	4.0e-12	6.4	2.8e-11	-0.9	0.84043	-0.9	0.83992	-1.8	0.96794	17
RELA	8.9	6.9e-21	8.3	3.5e-18	7.5	8.5e-15	-0.7	0.78668	-0.4	0.67143	-1.1	0.88305	13
SP1	4.7	6.8e-07	3.5	0.00013	2.6	0.00338	1.0	0.15222	0.7	0.22916	1.2	0.10234	33
TFAP2A	4.1	1.0e-05	2.9	0.00118	1.8	0.02925	-0.02	0.51006	-0.7	0.76218	-0.4	0.68439	<1

[1]Enrichments with p-values <0.001 are highlighted in grey; [2]TFs with RPKM <1 in mRNA were considered to be weakly or not expressed

B. Significantly enriched[1] TFBS in promoters of deacetylated and silenced genes

	genes induced after 1h LPS						genes deacetylated and silenced after 1h LPS						[2]RPKM
	2 fold		3 fold		5 fold		2 fold		3 fold		5 fold		Mo-DC
TFBS	z	p	z	p	z	p	z	p	z	p	z	p	mRNA
ELF5	1.5	0.05678	1.1	0.12202	1.4	0.06800	4.7	8.6e-07	4.5	2.0e-06	3.6	0.00010	<1
ETS1	0.4	0.32736	-0.7	0.77687	-0.3	0.63129	2.8	0.00184	3.0	0.00096	2.2	0.01083	17
FEV	1.5	0.06103	1.1	0.12125	0.2	0.38378	4.6	9.7e-07	3.8	4.7e-05	3.1	0.00072	<1
FOXC1	-0.4	0.66829	-1.1	0.88817	-1.1	0.87579	3.1	0.00064	2.6	0.00367	2.3	0.00932	<1
GABPA	2.7	0.00245	0.5	0.29199	-0.5	0.71052	3.6	0.00011	3.5	0.00019	2.4	0.00597	12
Myb	0.4	0.32290	0.2	0.40198	-0.5	0.72244	2.9	0.00117	2.3	0.00852	3.3	0.00043	<1
SPI1 (PU.1)	1.8	0.02517	1.4	0.06873	1.5	0.05428	5.5	7.4e-09	5.1	7.6e-08	4.8	5.9e-07	463
SPIB	1.7	0.03898	0.9	0.17594	0.9	0.16330	3.7	7.1e-05	3.9	3.4e-05	3.1	0.00067	<1

[1]Enrichments with p-values <0.001 are highlighted in grey; [2]TFs with RPKM <1 in mRNA were considered to be weakly or not expressed

Supplementary Table S3. Functions and genes affected by silencing

	RNA sequencing (RPKM)[1]			
	nascent transcripts		mRNA	
	DC	DC+LPS	DC	DC+LPS
**Antigen presentation**				
MR1	0.66	0.28	37.74	25.26
CD1A	20.29	5.72	441.79	310.61
CD1B	34.77	12.17	637.08	561.59
CD1E	10.45	2.95	244.03	192.42
CIITA	50.77	9.67	145.00	79.37
MARCH1	0.91	0.06	76.97	12.50
LNPEP	1.05	0.21	21.71	16.71
BTN3A1	1.78	0.56	11.26	7.86
**Receptor activity**				
PTPRE	13.73	6.6	258.02	219.61
FPR3	30.64	14.78	251.51	212.39
CSF3R	0.66	0.30	7.74	7.74
TRIM5	2.92	1.28	59.35	45.77
ITGB1	3.31	1.43	101.97	96.91
MR1	0.19	0.02	0.29	0.29
CR1	5.05	0.74	74.66	0.17
GPR82	0.31	0.13	1.69	0.05
CCR5	5.71	2.30	118.47	108.53
ASGR2	0.34	0.13	5.56	3.77
MSR1	0.001	0.00	6.69	6.43
LTB4R	8.78	2.90	11.45	6.11
RAMP1	7.82	2.51	206.92	178.35
TLR4	2.42	0.75	28.19	19.65
CSF2RB	8.37	2.37	120.78	100.07
GPR18	7.40	2.06	0.34	0.23
SPN	53.18	14.70	245.01	171.06
IL13RA1	1.59	0.41	68.58	54.29
ACVR1	0.50	0.13	8.67	10.95
CLEC4A	27.73	6.65	439.63	297.29
CD36	3.86	0.81	52.49	34.28
RARA	11.38	2.19	29.21	12.90
IFNGR1	9.43	1.79	215.71	127.36
IL21R	1.54	0.25	22.64	12.16
ANXA9	0.33	0.05	7.97	2.67
GFRA2	0.20	0.03	4.98	5.01
CCR1	5.05	0.74	74.66	0.17
RYK	1.70	0.22	16.49	13.34
SUCNR1	3.41	0.31	256.64	150.41
CSF1R	10.62	0.96	379.65	324.06
FCER1A	0.42	0.03	146.63	75.66
PTPRO	1.23	0.08	53.86	32.66
FZD5	0.71	0.04	2.047	0.99
FCER1G	3.32	0.14	258.03	127.06
DAB2	3.99	0.17	57.98	38.08
TLR1	1.55	0.06	7.28	13.10
LY96	1.82	0.07	49.36	36.60

**Endocytosis**				
ITGB1	3.31	1.43	101.97	96.91
MAPKAPK3	14.63	5.96	199.67	198.99
RAB7A	21.13	8.49	577.03	470.69
MSR1	0.001	0.00	6.69	6.43
ABCA7	1.47	0.55	8.12	6.50
GAPVD1	0.67	0.21	13.89	10.72
RAMP1	7.82	2.51	206.92	178.35
ALOX15	140.70	36.12	1157.52	1085.19
SMAP1	0.57	0.14	15.74	12.990
CD36	3.86	0.81	52.49	34.28
INPPL1	10.62	2.18	60.54	49.89
RARA	11.38	2.19	29.21	12.90
EPN1	2.51	0.46	22.60	17.65
CBL	6.62	1.04	32.71	23.58
SYK	7.41	1.16	118.07	81.94
WIPF1	20.84	3.17	75.69	65.88
PECAM1	8.17	1.18	134.92	86.11
CLEC10A	19.90	2.58	383.62	249.55
PIK3CA	1.16	0.13	17.18	13.80
MRC1	2.09	0.23	648.03	601.10
BIN2	0.99	0.10	38.46	28.19
EPN2	1.33	0.11	9.14	7.52
LDLRAP1	1.62	0.11	10.09	7.38
PIK3CG	1.21	0.07	17.80	11.76
FCER1G	3.32	0.14	258.03	127.06
DAB2	3.99	0.179	57.98	38.08
**Phagocytosis**				
CD302	4.83	2.41	56.46	46.17
RAB7A	21.13	8.49	577.03	470.69
ABCA7	1.47	0.55	8.12	6.50
ALOX15	140.70	36.12	1157.52	1085.19
CD36	3.86	0.81	52.49	34.28
RARA	11.38	2.19	29.21	12.90
SYK	7.41	1.16	118.07	81.94
WIPF1	20.84	3.17	75.69	65.88
PECAM1	8.17	1.18	134.92	86.11
PIK3CA	1.16	0.13	17.18	13.80
BIN2	0.99	0.10	38.46	28.19
FCER1G	3.32	0.14	258.03	127.06
**Vesicular transport**				
PDPK1	1.62	0.42	29.62	19.81
ALOX15	140.70	36.12	1157.52	1085.19
SMAP1	0.57	0.14	15.74	12.99
CD36	3.86	0.81	52.49	34.28
CBL	6.62	1.04	32.71	23.58
SYK	7.41	1.16	118.07	81.94
HMOX1	1.17	0.09	155.81	100.988
FCER1A	0.42	0.03	146.63	75.66
LDLRAP1	1.62	0.11	10.09	7.38
FCER1G	3.32	0.14	258.03	127.06
DAB2	3.99	0.17	57.98	38.08

**Immune response signaling pathway**				
TRIM5	2.92	1.28	59.35	45.77
MAPKAPK3	14.63	5.96	199.67	198.99
FOS	6.84	2.73	9.93	8.71
TNRC6B	2.60	0.86	4.44	3.67
UBE2D1	0.25	0.08	13.18	12.21
BTN3A1	1.78	0.56	11.26	7.86
TLR4	2.42	0.75	28.19	19.65
CD226	6.15	1.64	44.24	11.24
PDPK1	1.62	0.42	29.62	19.81
CD36	3.86	0.81	52.49	34.28
CNPY3	3.77	0.64	64.05	48.99
SYK	7.41	1.16	118.07	81.94
WIPF1	20.84	3.17	75.69	65.88
PIK3CA	1.16	0.13	17.18	13.80
NFATC3	4.40	0.48	45.65	38.46
FYB	1.97	0.15	23.55	25.21
FCER1A	0.42	0.03	146.63	75.66
NOD1	5.33	0.37	20.21	7.89
FCER1G	3.32	0.14	258.03	127.06
TLR1	1.55	0.06	7.28	13.10
LY96	1.82	0.07	49.36	36.60
MAP2K6	0.44	0.01	24.97	13.76
**Icosanoid metabolic process**				
GGT7	1.48	0.41	5.29	3.96
ALOX15	140.70	36.12	1157.52	1085.19
PTGS1	16.88	3.81	147.05	98.70
DPEP2	15.13	2.79	35.70	21.66
SYK	7.41	1.16	118.07	81.94
LTA4H	0.21	0.02	35.026	32.08
FCER1A	0.42	0.03	146.63	75.66
**Apoptotic signaling pathways**				
HIPK1	2.10	0.79	27.24	19.95
RNF7	1.15	0.43	28.78	19.98
MADD	2.16	0.78	22.00	18.09
ZNF385A	3.74	1.18	44.39	46.63
TMEM109	0.17	0.05	34.83	26.72
ATM	2.62	0.76	16.53	15.48
PDPK1	1.62	0.42	29.62	19.81
ACVR1	0.50	0.13	8.67	10.95
CIDEB	10.05	2.56	59.57	39.64
WFS1	0.66	0.15	13.14	11.06
KDM1A	0.61	0.11	24.30	23.95
HMOX1	1.17	0.09	155.81	100.98
IFI16	4.64	0.22	94.06	51.86
**Leukocyte migration**				
CSF3R	0.66	0.30	7.74	7.74
ITGB1	3.31	1.43	101.97	96.91
CCR5	5.71	2.30	118.47	108.53
LYST	6.21	2.37	20.87	17.10
SPN	53.18	14.70	245.01	171.06
AMICA1	9.38	2.24	196.98	128.53

DOK2	18.07	3.85	195.16	118.68
SYK	7.41	1.16	118.07	81.94
CCR1	5.05	0.74	74.66	0.17
PECAM1	8.17	1.18	134.92	86.11
ADAM10	0.99	0.13	53.69	40.95
SELPLG	1.84	0.23	62.22	83.32
PIK3CA	1.16	0.13	17.18	13.80
HMOX1	1.17	0.09	155.81	100.98
PTPRO	1.23	0.08	53.86	32.66
PIK3CG	1.21	0.07	17.80	11.76
SLC7A8	3.69	0.16	63.49	44.45
FCER1G	3.32	0.14	258.03	127.06
CKLF	12.17	0.47	177.02	48.42
**Cytokine production**				
OTUD5	1.82	0.89	27.31	22.34
MR1	0.66	0.28	37.74	25.26
LRRFIP1	16.96	6.36	121.58	97.30
BTN3A1	1.78	0.56	11.26	7.86
TLR4	2.42	0.75	28.19	19.65
SPN	53.18	14.70	245.01	171.06
ACP5	7.86	2.04	658.78	649.79
BTN3A2	0.35	0.08	12.40	9.36
CD36	3.86	0.81	52.49	34.28
RARA	11.38	2.19	29.21	12.90
SYK	7.41	1.16	118.07	81.94
CARD8	3.56	0.54	18.26	12.30
GBA	1.05	0.12	36.92	37.75
FURIN	25.25	2.63	401.92	245.56
CSF1R	10.62	0.96	379.65	324.06
HMOX1	1.17	0.09	155.81	100.98
FCER1A	0.42	0.03	146.63	75.66
NOD1	5.33	0.37	20.21	7.89
FZD5	0.71	0.04	2.04	0.99
IFI16	4.64	0.22	94.06	51.86
FCER1G	3.32	0.14	258.03	127.06
TLR1	1.55	0.06	7.28	13.10
GPR120	1.81	0.02	25.08	7.83

[1]average RPKM of three independent experiments is shown
